# Diagnosis and management of vascular Ehlers-Danlos syndrome: Experience of the UK national diagnostic service, Sheffield

**DOI:** 10.1038/s41431-023-01343-7

**Published:** 2023-03-29

**Authors:** Jessica M. Bowen, Monica Hernandez, Diana S. Johnson, Claire Green, Tammy Kammin, Duncan Baker, Sylvia Keigwin, Seiko Makino, Naomi Taylor, Oliver Watson, Nigel M. Wheeldon, Glenda J. Sobey

**Affiliations:** 1 Ehlers-Danlos Syndrome National Diagnostic Service, Sheffield Children’s Hospital NHS Foundation Trust, OPD2 Department, Northern General Hospital, Herries Road, Sheffield, S5 7AU UK; 2 Health Economics and Decision Science, ScHARR, University of Sheffield, Regent Court, 30 Regent Street, Sheffield, S1 4DA UK; 3 Sheffield Diagnostic Genetics Service, Sheffield Children’s Hospital NHS Foundation Trust, Sheffield, S10 2TH UK; 4 South Yorkshire Regional Inherited Cardiac Conditions Service, Sheffield Teaching Hospitals NHS Foundation Trust, Northern General Hospital, Herries Road, Sheffield, S5 7AU UK

**Keywords:** Aortic diseases, Preventive medicine, Genetic testing

## Abstract

The UK National Diagnostic Service for Ehlers-Danlos Syndromes (EDS) was established in 2009 for the rare types of EDS. Vascular EDS (vEDS) is an inherited connective tissue disorder caused by pathogenic variants in the *COL3A1* gene. Associated tissue fragility affects multiple organ systems, increasing the risk of blood vessel dissection and rupture, with potentially fatal consequences. The diagnosis of vEDS has improved with advances in genetic testing, however this is most often suspected following an acute event. We provide data on the clinical features of vEDS for 180 patients (full cohort) seen in our service with confirmed molecular diagnoses. Increased awareness of this rare condition will prompt genetic testing essential to confirm the diagnosis. Outcomes are improved by early diagnosis followed by appropriate management. Fragile connective tissues make invasive procedures potentially dangerous, particularly in an emergency setting. Lifestyle advice from a young age can help acceptance and understanding of the diagnosis and inform choices. There is currently limited evidence for the use of drug therapy to reduce vascular events. We report on the incidence of vascular events in 126 patients (statistical analysis cohort) in our care and the use of medication. Our retrospective data showed that those patients on a long-term angiotensin II receptor blocker and/or beta-blocker had fewer vascular events than those not on cardiac medication who received the same lifestyle and emergency care advice.

## Introduction

Vascular Ehlers-Danlos syndrome (vEDS) is rare connective tissue disorder caused by pathogenic variants in the *COL3A1* gene. It is an autosomal dominant condition for which genetic testing is required for a definitive diagnosis [1]. There are 13 types of EDS described in the 2017 diagnostic criteria [2]. VEDS is characterised by fragile connective tissues caused by abnormal type III collagen. Blood vessel dissection and rupture as well as hollow organ rupture are potentially fatal consequences of vEDS. It is thought to affect approximately 2 in 100,000 individuals and this accounts for less than 5% of EDS diagnoses [3].

There have been few large vEDS patient cohorts published [4, 5]. Other studies have looked at pregnancy risks, gastrointestinal manifestations, surgical outcomes and medication [1, 6–10]. Evidence suggests that having a correct diagnosis made, with appropriate clinical management and long-term follow-up improves survival for vEDS patients [8, 10]. It is recognised that the diagnosis is often made following an adverse event [11].

The 2017 classification lists major and minor criteria that are suggestive of vEDS and should lead to diagnostic testing [2]. It is accepted that even for experienced clinicians the diagnosis of vEDS requires molecular confirmation to differentiate from conditions with a similar presentation. A vEDS diagnosis is confirmed once a pathogenic variant is found on one allele of *COL3A1*.

Management guidelines are still lacking for vEDS, despite increasing evidence to support specific pregnancy and surgical management [6, 8]. In the absence of major clinical trials, current drug therapies are largely based on extrapolation of data in other genetic disorders affecting the vasculature [12]. A trial using celiprolol was carried out prior to molecular confirmation of a diagnosis in all participants. As a result, this study included patients in whom the diagnosis was not molecularly proven to be vEDS [13]. A long-term observational study of vEDS patients treated with celiprolol reported an improved survival rate but could not determine to what extent this was due to the overall medical care rather than being due to this specific medication [10]. Hence there is still limited evidence to demonstrate that medication reduces vascular events for vEDS patients, or which medications should be offered.

The UK EDS National Diagnostic Service was established in 2009 to improve diagnosis of the rare types of EDS. The service is based at two locations, Sheffield and London. Over 2000 patients and families have now been seen by the clinic in Sheffield, including over 200 people diagnosed with vEDS.

We present the experience acquired in the Sheffield centre over a period of 12 years, collecting data from diagnosis until May 2021. The data was analysed with the aim of adding to international datasets on vEDS. The main results are included in this paper, with additional results and resources provided in an online supplement.

## Subjects and methods

All vEDS patients were seen by consultants D.J. or G.S. for clinical assessment. Confirmation of the diagnosis was made in all cases by genetic testing.

**Fig. 1:**
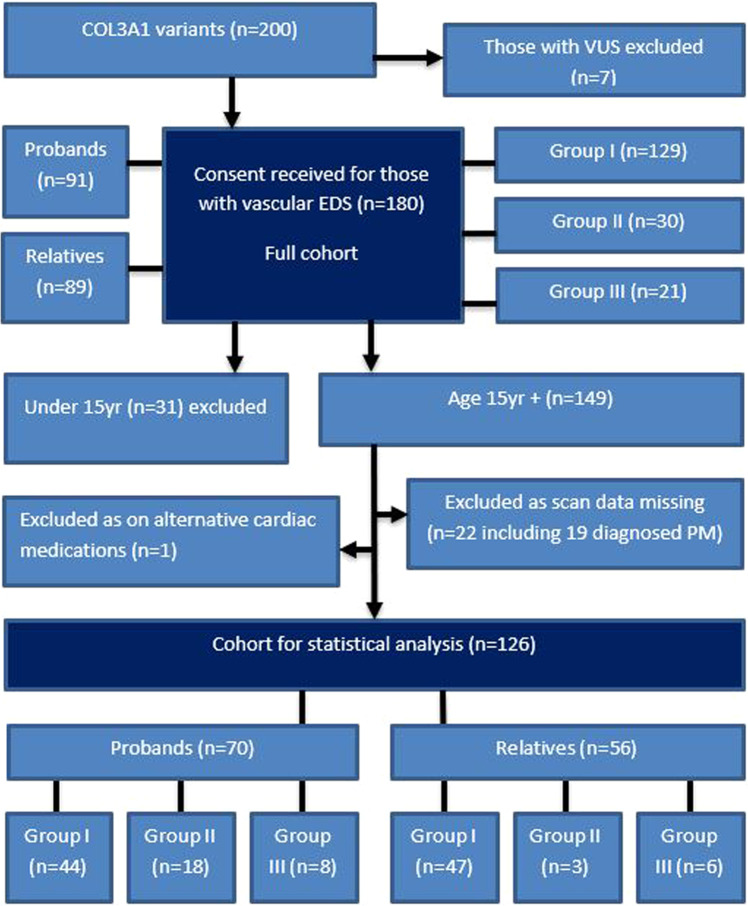
Study identification, inclusion and exclusion flow chart for the statistical analysis cohort. Group I = glycine substitutions within the triple helix, Group II = splice site variants and in frame deletions and duplications, Group III = variants causing haploinsufficiency [5]. Those age under 15 excluded to allow comparison with previous cohort [10]. (PM = post mortem).

Molecular genetic testing was carried out by Sheffield Diagnostic Genetics Service accredited laboratory using Sanger sequencing, MLPA and next generation sequencing. Variant interpretation was carried out by Clinical Scientists according to the ACMG and ACGS guidelines [14]. Patients with variants of unknown significance (VUS) in COL3A1 were excluded. In line with previous studies the molecular results were grouped into: glycine substitutions within the triple helix (Group I), splice site variants, in-frame deletions and duplications (Group II), and variants causing haploinsufficiency (Group III) Fig. 1.

All patients testing positive for vEDS were reviewed and received comprehensive advice on the management of vEDS. This included lifestyle advice, guidance on when to seek medical attention and a discussion of activities that may increase risk. All patients are given emergency information cards to present to any medical professional they consult (Online Supplement Fig. 1).

Following diagnosis all patients (aged 13 and over) are invited to be followed up in our joint vEDS Inherited Cardiac Conditions clinic. Discussion of medication and imaging is undertaken at the first appointment. A baseline magnetic resonance angiogram (MRA) is offered. This assesses the whole of the thoracic and abdominal aorta, cervical vessels to include the circle of Willis and extends to the femoral arteries. This is repeated annually, or more frequently when there are clinical concerns. We discuss the options of medications including beta blockers (BB) and angiotensin II receptor blockers (ARB), explaining the limitations of current evidence for this rare condition. In the absence of large trial data and extrapolating from data in other genetic diseases affecting the vasculature, we offer combined therapy with an ARB and BB. Patients are made aware of the limitations of this approach, until such time as more trial data is available. We start with a low dose of either BB or ARB (most often bisoprolol and losartan, see Online Supplementary Fig. 2) before introducing the second medication. Doses are uptitrated, with an aim to get to a full dose of both, dependant on patient tolerance. Patients who decided against taking medication are included here as a control group, as they received the same lifestyle advice and imaging follow up. Some patients took just one medication, due to side effects, contra-indications or personal preference.

In total 180 patients with molecularly confirmed vEDS consented to publication, 97 females and 83 males (Online Supplementary Table 1). Clinical data, medical history, medications, and scan history were recorded at diagnosis and at each follow-up contact, to ensure that all vEDS-related events were included. These were documented contemporaneously. Ethical approval was granted for patient data to be reviewed and analysed. The data was anonymised before analysis by the University of Sheffield, School of Health and Related Research (ScHARR).

### Statistical analysis

The analysis was designed to allow comparison with the previous long-term observational study by Frank et al. 2019 [10]. For this reason, patients were included from age 15. Baseline data was collected when the patient was first seen by the service. Vascular events including those from scan results were scored using the empirical scoring system developed by Frank et al. 2019 which assigns a score depending on the clinical consequence of each vascular event to produce a clinical progression score by comparing the final vascular event score to the patient’s score at baseline (Online Supplementary Table 2). Medication was logged in a timeline against the vascular event score, to compare clinical progression and survival with drug treatment. Quantitative variables were presented as a median, interquartile range and count as appropriate and qualitative variables as count and percentage. Kruskal-Wallis and Fisher exact tests were used for comparisons of quantitative and qualitative variables respectively. The Kaplan-Meier estimate of the survivor function was used to examine overall survival and compare across treatments. The equality of survivor functions was examined using a log-rank test. All statistical analyses were carried out using Stata version 17.0 [15].

## Results

### Full cohort

The full cohort of 180 patients includes 95 families with an age range from 2 to 80 years and is evenly split between males and females (83:97). The molecular results of all 180 patients are included in the online supplement (Online Supplementary Table 1). The vast majority (*n* = 129) have Group I variants as expected, since glycine substitutions are known to be the predominant cause of vEDS (Fig. 1). 30 patients have Group II variants, the majority being splice site variants (*n* = 28) and just 2 with in-frame deletions/duplications. 21 patients had Group III variants, including 2 patients who died of aortic dissection age 44 and 55, prior to being seen by the service. In total 17 patients were diagnosed post-mortem (Online Supplementary Table 3).

### Reason for diagnosis

The majority of people in our cohort were diagnosed following a vEDS related event themselves or in the family. 49 were diagnosed following a vascular event, 21 were diagnosed following an organ rupture and 4 had both an organ rupture and a vascular event prior to their diagnosis. 78 had a relative already diagnosed with vEDS. 8 had a family history suggestive of vEDS, in addition to clinical features, which led to them being tested.

20 patients were diagnosed based on their clinical appearance alone. Of these 20: 6 had talipes, 2 had amniotic bands, 2 had distal contractures, 18 had thin skin, 16 easy bruising, 11 acrogeria, all had prominent hollow eyes. 4/20 patients were diagnosed by microarray which showed a de novo microdeletion involving *COL3A1*. 8/20 patients were found to have de novo sequence variants. Another 4/20 were considered likely to be de novo, whilst 4/20 were found to have inherited the condition.

### Clinical features

Looking at the full cohort, for those where features were assessed 94.6% had prominent/hollow eyes, 75.3% delicate midface, 85.2% attached earlobes/lobeless ears. Acrogeria was observed in 41.8% and thinning hair in 29.7%. Easy bruising and thin skin were seen in the majority (83% and 74% respectively) but not all patients. Premature birth was recorded in 27%, far higher than the current UK premature birth rate of 8%. Of those where the Beighton score was recorded 64.1% had small joint hypermobility of their fingers, fewer (32.8%) had generalised hypermobility with a score of 5 + .

There are additional rarer features that may help to make earlier diagnoses. Early onset varicose veins were seen in 15% who had varicose veins under 30, excluding those that started following pregnancy. Talipes was seen in 13%. This was significant and not correctable with physio alone with 15/21 needing surgery. Distal contractures were seen in 4%. Of the 7 people who had distal contractures, 2 had contractures of the Achilles tendon, 3 had contractures of the hands and feet. These were all progressive and resistant to treatment. 2 people had milder 5th finger contractures. 4 people had clinical findings attributed to amniotic bands [16].

Keratoconus previously reported in association with vEDS only occurred in 2 people in our cohort (1.11%). One person in our cohort had congenital hip dysplasia (0.55%).

### Vascular events

72 patients (40%) had a total of 165 vascular events (Online Supplementary Table 9). Of these 36 (50%) had a single event and the maximum was 9 vascular events in one patient. The youngest vascular event was in a male patient who had an axillary artery rupture age 12. There were 25 fatal vascular events, and 13 of these were aortic dissections.

6 patients (3.33%) had carotid cavernous fistula, all were female aged 29 to 55 years. Haemoptysis was reported by 5 patients (2.77%), once in association with haemothorax.

108 patients, aged 2–80 yrs, had no vascular events. Their median age was 22 and IQR was 13–36.

### Organ ruptures

25 patients (13.88%) had bowel perforation. The majority of these were under the age of 30 years, age range 9–54 years. The 54 year old also had diverticulitis. Of those with Group III variants there was just one sigmoid colon perforation, in a female age 46. There are two peak ages for bowel perforation, mid-teens and early twenties (Online Supplementary Fig. 3). In one family three family members all had bowel perforations with successful reversal surgery. However, 7 patients had further bowel perforation, including one patient who perforated her colon age 12 years despite already having a colostomy following rectal perforation age 11 years.

10 patients (5.55%) had pneumothorax, the youngest just one day old. Eight of these occurred before the diagnosis of vEDS was made. 4 patients had ruptured spleens. No one in our cohort had a uterine rupture.

### Pregnancy

53 women in the cohort had a combined total of 114 pregnancies. 1 patient had IVF which was unsuccessful but without vEDS related events. In-line with previous studies significant bleeding and tearing at delivery were commonly reported on patient history. Excluding the women with Group III variants, 10% of women had a life-threatening or fatal vascular event in pregnancy or the perinatal period (5/48), which accounted for 5 significant vascular events in 104 pregnancies (4.8%). 3 fatalities were all in first pregnancies; 1 spontaneous iliac artery dissection during labour caused cerebral palsy in combination with vEDS for the child who was delivered by posthumous caesarean section, 1 patient died 7 days post emergency c-section with aortic dissection and 1 patient had a fatal dissection of the thoracic aorta the day after surgical termination of pregnancy at 10 weeks. 1 patient survived a left iliac artery dissection 9 days after delivery of her second child, followed by a bowel perforation 6 days later and is doing well 24 years later. One patient survived a spontaneous coronary artery dissection (SCAD) at 29 weeks gestation.

9 babies were successfully delivered following vEDS diagnosis to 8 women (2 with Group III variants). They all had early delivery by planned c-section under general anaesthetic in a tertiary hospital. One planned c-section was brought forward due to maternal pancreatitis (patient had a Group III variant) and one patient had premature rupture of membranes (PROM) 24 h before planned c-section. Details of pregnancy management were outlined in a poster (Online Supplementary Fig. 5).

### Statistical analysis cohort

126 patients were included in the statistical analysis, 54 patients were excluded (Fig. 1).

Baseline characteristics according to proband /relative status and final treatment are shown in Tables 1, 2. There were 77 females and 49 males diagnosed at a median age of 29 years (20, 44.25 IQR). The split between probands and relatives is 70:56, similar to the French cohort and not significantly different (Fisher’s exact *p*-value = 0.216). Consistent with previous cohorts, the majority have Group I variants (91) and smaller numbers have Group II (21) and Group III variants (14). The proportion of patients in Group II is significantly lower than seen in the French cohort, but we had a similar proportion of probands to relatives (18:3) within this group. It is not clear why we have fewer patients with Group II variants but may just reflect the relatively small numbers involved. The smallest group is of those with Group III variants, which may reflect this patient group being under ascertained due to the potential for these variants to cause on average a later onset phenotype. The small number in this group will limit any bias from this in our study. At baseline, 43 (34.13%) patients had already experienced a total of 71 vascular events, 19 bowel perforation, 5 pneumothorax and 2 ruptured spleen. The longest follow up was over 10 years with a median follow up of 3.8 years, reflecting the increasing number of referrals to the service over the years.

**Table 1:** Baseline characteristics of statistical analysis cohort according to proband/relative status.

Characteristics	Relatives		Proband		*p*-value
n	56		70		
**Sex**					
Female	39(69.64)		38(54.29)		0.0973
Male	17(30.36)		32(45.71)		
Age at diagnosis	26.5(20,44)		32(19.75,44.5)		0.6166
**Type of variant**		**Age at baseline**		**Age at baseline**	
Group I	47(83.93)	29(20,45)	44(62.86)	35(21,46)	0.0060
Group II	3(5.36)	28(23,52)	18(25.71)	29(15,43)	
Group III	6(10.71)	23(17,40)	8(11.43)	45(34,54)	
**Medical history**					
**Arterial events**					
Patients	5(8.93)		38(54.29)		0.0000
No. events	5		67		
**Spleen events**					
Patients	1(1.79)		1(1.43)		0.8718
No. events	1		1		
**Bowel events**					
Patients	4(7.14)		15(21.43)		0.0430
No. events	5		19		
**Pneumothorax events**					
Patients	1(1.79)		4(5.71)		0.2780
No. events	2		4		
**Pulmonary events**					
Patients	2(3.57)		7(10)		0.1636
No. events	2		8		
**Overall events**					
Patients	13(23.21)		53(75.71)		0.0000
No. events	15		99		
CA score	0(0,0)		1.5(0,4.25)		0.0000
[min,max]	[0,10]		[0,22]		
FU duration	2.65(1.96,4.52)		5.22(2.87,7.16)		0.0023
[min,max]	[0.27,10.56]		[0.85,10]		

Values are *n*(%) or median (interquartile range). Fisher exact test for qualitative variables and Kruskal-Wallis test for quantitative variables (celiprolol group is excluded from the test due to low numbers).

**Table 2:** Baseline characteristics according to final treatment groups.

	All	ARB	BB	BB&ARB	celi	celi&ARB	no treatm	*p*-value
n	126	18	9	73	2	9	15	
**Sex**								
Female	77(61.11)	14(77.78)	6(66.67)	38(52.05)	2(100)	5(55.56)	12(80)	0.1351
Male	49(38.89)	4(22.22)	3(33.33)	35(47.95)	0(0)	4(44.44)	3(20)	
Age at diagnosis	29(20,44.25)	30(15.5,47.75)	28(21,40.5)	30(18.5,43)	25.5(24,27)	36(30,49.5)	25(18,46)	0.6156
**Status**								
Relative	56(44.44)	10(55.56)	2(22.22)	30(41.1)	1(50)	4(44.44)	9(60)	0.3628
Proband	70(55.56)	8(44.44)	7(77.78)	43(58.9)	1(50)	5(55.56)	6(40)	
**Type of variant**								
Group I	91(72.22)	10(55.56)	8(88.89)	52(71.23)	2(100.00)	7(77.78)	12(80.00)	0.6401
Group II	21(16.67)	4(22.22)	1(11.11)	12(16.44)	0(0)	1(11.11)	3(20)	
Group III	14(11.11)	4(22.22)	0(0)	9(12.33)	0(0)	1(11.11)	0(0)	
**Medical history**								
**Arterial events**								
Patients	43(34.13)	5(27.78)	3(33.33)	27(36.99)	0(0)	5(55.56)	3(20)	0.1265
No. events	72	7	3	40	0	19	3	
**Spleen events**								
Patients	2(1.59)	1(5.56)	0(0)	1(1.37)	0(0)	0(0)	0(0)	0.6785
No. events	2	1	0	1	0	0	0	
**Bowel events**								
Patients	19(15.08)	3(16.67)	4(44.44)	7(9.59)	1(50)	2(22.22)	2(13.33)	0.0830
No. events	24	3	5	9	2	2	3	
**Pneumothorax events**								
Patients	5(3.97)	1(5.56)	1(11.11)	3(4.11)	0(0)	0(0)	0(0)	0.6879
No. events	6	2	1	3	0	0	0	
**Pulmonary events**								
Patients	9(7.14)	2(11.11)	1(11.11)	4(5.48)	0(0)	2(22.22)	0(0)	0.2931
No. events	10	2	1	5	0	2	0	
**Overall**								
Patients	66(52.38)	10(55.56)	6(66.67)	38(52.05)	1(50)	6(66.67)	5(33.33)	0.1036
No. events	114	15	10	58	2	23	6	
CA score^a^	0(0,2)	0(0,1)	0(0,1)	0(0,2)	0(0,0)	3(0,6.5)	0(0,0)	0.1937
[min/max]	[0,22]	[0,3]	[0,4]	[0,22]	[0,0]	[0,11]	[0,10]	
FU duration	3.81(2.08,6.39)	2.83(1.70,4.21)	2.27(1.20,5.80)	4.67(2.46,6.91)	1.01(1.01)	2.06(2.04,3.79)	3.44(2.29,5.22)	0.0857
[min/max]	[0.27,10.56]	[0.97,8.96]	[1.04,9.33]	[0.27,10.56]	[1.01,1.03]	[1.41,7.10]	[1.13,9.54]	

Values are *n*(%) or median (interquartile range). Fisher exact test for qualitative variables and Kruskal-Wallis test for quantitative variables (celiprolol group is excluded from the test due to low numbers.)

^a^Clinical Arterial score.

As expected, probands showed a higher proportion of previous clinical events. 75.71% of probands had prior vEDS-related events compared to 23.21% of relatives (Table 1). The probands were 32:38 male to female, while the relatives are majority female (39) to male (17). Of the probands 54% were found to have inherited the variant from a parent and 20% were proven de novo. A further 26% could not be confirmed as de novo, but showed no clear family history of vEDS (Online Supplementary Table 4).

### Treatment groups

A small number (*n* = 28; 22.22%) of patients were already on treatment when they were referred to the service (Online Supplementary Table 5). We grouped patients according to the treatment they were taking at the end of follow-up to create 6 treatment groups: BB, ARB, BB&ARB, celiprolol, celiprolol&ARB, and no treatment. Our BB and BB&ARB groups included all BBs except celiprolol, to analyse this separately. We felt this would be helpful, to compare against the French cohort who are on celiprolol [10]. Our no treatment group contained only people on no cardiac medication, so allowed us to have a control group under the same service, getting the same lifestyle advice, emergency medical information and follow up imaging. 15 patients remained on no treatment and make up the control group Table 3). There was no significant difference between the final treatment groups in relation to age, sex, type of variant or prior history of clinical events (Table 2).

**Table 3:** Clinical Outcomes during follow-up according to treatment.

	All	ARB	BB	BB&ARB	celi	celi&ARB	no treatm	*p*-value
*n*	126	18	9	73	2	9	15	
**Arterial events**								
Patients	26(20.63)	2(11.11)	2(22.22)	15(20.55)	1(50.00)	1(11.11)	5(33.33)	
No. events	39	3	3	26	1	1	6	0.5945
Events 5 yrs	1.25(0.78,4.04)	5.60(0.86,10.34)	2.47(0.54,4.40)	1.31(0.67,3.02)	4.87(4.87,4.87)	1.11(1.11,1.11)	1.15(0.77,3.53)	0.9784
**Bowel events**								
Patients	3(2.38)	1(5.56)	0(0.00)	2(2.74)	0(0.00)	0(0.00)	0(0.00)	
No. events	3	1	0	2	0	0	0	0.8111
Events 5 years	0.86(0.72,1.86)	2.18(2.18,2.18)	-	0.83(0.68,0.90)	-	-	-	0.2207
**Pneumothorax events**								
Patients	1(0.79)	0(0.00)	1(11.11)	0(0.00)	0(0.00)	0(0.00)	0(0.00)	
No. events	1	0	1	0	0	0	0	0.0124
Events 5 years	0.54(0.54,0.54)	-	0.54(0.54,0.54)	-	-	-	-	0.0000
**Pulmonary events**								
Patients	5(3.97)	0(0.00)	2(22.22)	2(2.74)	0(0.00)	1(11.11)	0(0.00)	
No. events	5	0	2	2	0	1	0	0.0331
Events 5 years	0.83(0.50,1.66)	-	0.70(0.54,0.86)	0.65(0.47,0.83)	-	2.45(2.45,2.45)	-	0.3012
**All events**								
Patients	31(24.60)	3(16.67)	3(33.33)	17(23.29)	1(50.00)	2(22.22)	5(33.33)	
No. events	48	4	6	29	1	2	6	0.7654
Events 5 years	1.61(0.86,3.02)	2.18(0.86,10.34)	1.61(0.86,4.40)	1.31(0.74,2.70)	4.87(4.87,4.87)	1.78(1.11,2.45)	1.15(0.77,3.53)	0.9456
CA score base	0.00(0.00,2.00)	0.00(0.00,1.00)	0.00(0.00,1.00)	0.00(0.00,2.00)	0.00(0.00,0.00)	3.00(0.00,6.50)	0.00(0.00,0.00)	
CA score end	0.00(0.00,4.00)	0.00(0.00,1.50)	0.00(0.00,1.50)	0.00(0.00,4.00)	5.00(0.00,10.00)	4.00(0.00,9.00)	0.00(0.00,10.00)	
CA score base^a^	2(0,5)	2(1,3)	1(0,1)	2(1,7)	0(0,0)	5(5,5)	0(0,8)	
CA score end^a^	10(4,12)	9(6,11)	2(1,2)	6(3,12)	10(10,10)	10(10,10)	12(10,16)	
Death	10(7.94)	1(5.56)	0(0)	4(5.48)	1(50)	0(0)	4(27)	
Death (vEDSrelated)	8(6.35)	0(0)	0(0)	3(4.11)	1(50)	0(0)	4(27)	

Values are *n*(%) or median(interquartile range). Fisher exact test for qualitative variables and Kruskal-Wallis test for quantitative variables(celiprolol groups excluded from the test due to low numbers).

^a^Sample of those with arterial events during follow-up.

Of those untreated at baseline, 83 went on to medication (Online Supplementary Table 5). The majority of patients (57%) transitioned from no treatment to BB&ARB which reflects the recommendations of our service. Those on BB&ARB (*n* = 73) had the longest median follow-up of 4.67 years (min 0.27 and max 10.56). The next largest group was those on an ARB alone (*n* = 18). This group includes those unable to take BB due to contra-indications, e.g asthma, and those who are planning to add BB at a later date. There were only small numbers in the BB, celiprolol and celiprolol&ARB groups (*n* = 9, 2, and 9 respectively). Of the 15 patients that remained on no treatment, one tried an ARB but had to stop due to an allergic reaction.

### Vascular clinical progression score

The majority of our patients (*n* = 88; 69.84%) started in the very low-scoring group (Online Table 6). At the end of follow up most of our patients remained in their original progression group, indicating high clinical stability. Only 15 (11.90%) moved to a higher progression group by the end of follow-up and of those, 8 moved up by a single progression group. The number of patients on treatment who progressed was 10 out of 111 (9.01%) and 5 out of 15 (33.33%) in the untreated group (Fisher’s exact = 0.018). Only 6 out of 73 (8.22%) of those on BB&ARB progressed. Furthermore, 4 out of 111 (3.60%) in the treated and 3 out of 15 (20%) in the untreated group moved up two progression groups, indicating that those on treatment showed less clinical progression than the untreated group (Online Supplementary Table 7).

### Survival

92% (*n* = 116, 71 female/45 male) of patients were alive at the end of follow-up, aged 15–76 years (Online Supplementary Table 8). 10 patients died during follow-up, including two cancer-related deaths. These two patients were on ARB and BB&ARB when they died and are excluded from the discussion below, to reflect survival in relation to vEDS and the impact of medication. Eight patients had vEDS-related deaths during the study (4 male, 4 female, median age at death 26; interquartile range 19.25–30.5) (Table 4). Abdominal or thoracic aortic dissections/ruptures were the main cause of death (*n* = 5). The other 3 patients who died had blood vessel ruptures causing bleeding into the abdomen (Table 4). Of the 4 people who died while on treatment, 3 were on BB&ARB (*n* = 73). Of these 3 patients, 1 died very shortly after starting medication (just 3 months) and 2 already had very high vascular scores at baseline having both had life-threatening vascular events before diagnosis. One of these patients had a thoracic aortic dissection causing paraplegia at age 47 and died at age 51. The other patient lost their arm following a brachial artery rupture at age 12, had a right iliac artery dissection in the same year and lived another 8 years with medication. The fourth patient who died while on treatment was on celiprolol and died from their first arterial event after 12.3 months of treatment.

**Table 4:** Characteristics and causes of death in the 10 patients who died during follow up.

								Time under follow-up on treatment or with no treatment, in months (treatment sequence)					
Sex	Age at death	Proband/ Relative	vEDS variant	Follow up (years)	CA Score (1^st^seen)	CA Score (endFU)	Cause of death	ARB	BB	BB&ARB	celi	celi&ARB	no treatment
M	24	P	Group I	1.13	10	20	Ruptured renal artery aneurysm	-	-	-	-	-	13.5
M	29	R	Group I	4.34	0	10	Ruptured thoracic aorta	-	-	-	-	-	52.1
F	31	P	Group I	4.93	0	10	Dissection of thoracic aorta	-	-	-	-	-	59.1
F	19	P	Group II	3.81	0	12	Aortic dissection	-	-	-	-	-	45.8
F	28	P	Group I	1.03	0	10	Renal infarction, infracolic haematoma	-	-	-	12.3	-	
F	51	P	Group I	2.61	10	20	Vessel rupture in abdomen	-	-	31.3	-	-	
M	19	R	Group I	0.27	0	10	Aortic dissection	-	-	3.2	-	-	
F	55	R	Group I	2.46	1	1	Cancer^a^	-	-	27.4(2)	-	-	2.1(1)
F	76	R	Group III	1.99	0	0	Lung cancer^a^	23.8(2)	-	-	-	-	0.1(1)
M	20	P	Group I	5.30	7	17	Aortic dissection	-	31.1(1)	32.5(2)	-	-	

^a^Highlighted:death unrelated to vEDS.

Despite the small size of the no treatment group (*n* = 15), this group had the highest number of vEDS-related deaths (*n* = 4), 26.7% compared to 3.6% of those on treatment. 2 of these patients died following their first vascular event, both were considering starting (but not on) medication. Another patient had previously had bilateral carotid artery aneurysms identified on scan and had deferred starting medication for personal reasons. The fourth patient had previously had a life-threatening vascular event with a ruptured splenic artery aneurysm that was successfully coiled. They died following a ruptured renal artery aneurysm.

**Fig. 2:**
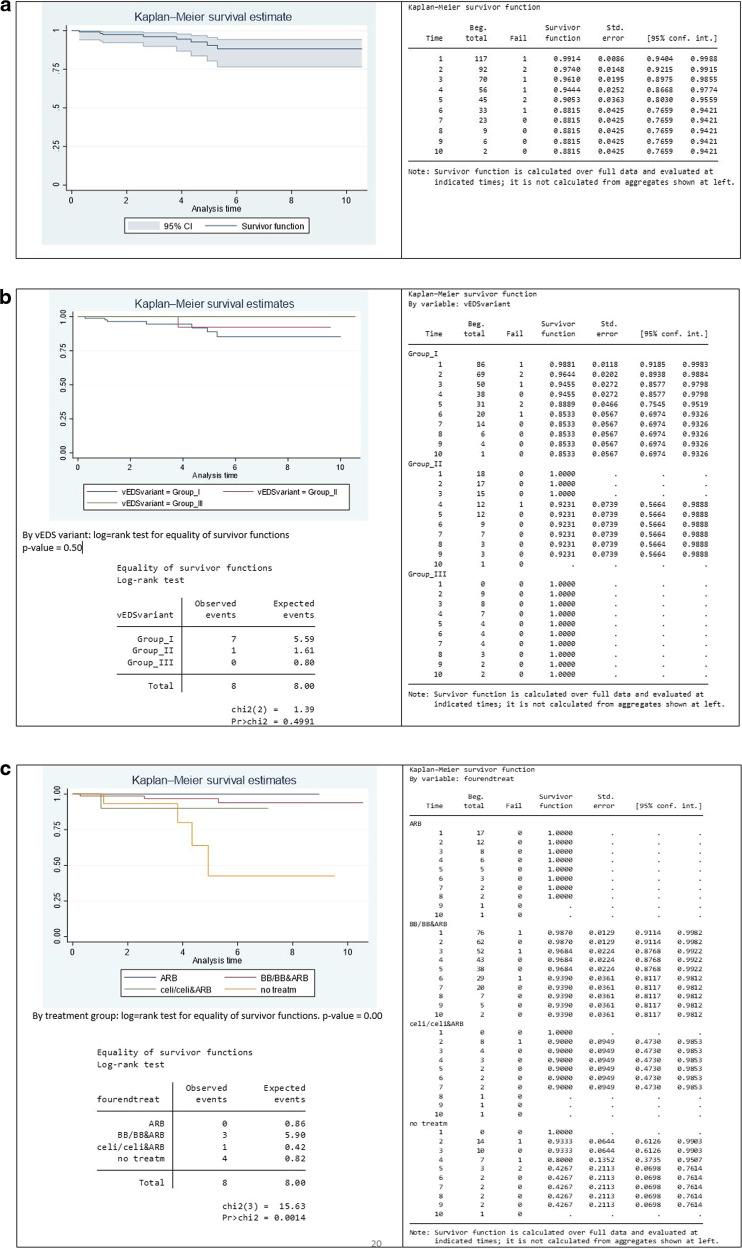

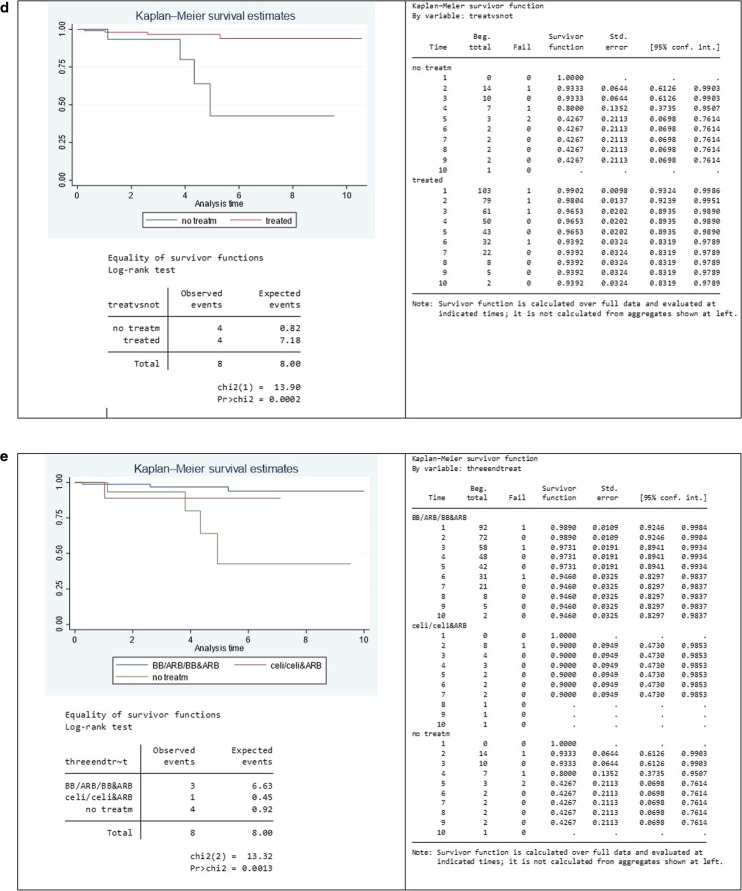
Survival Graphs. **a** Overall patient survival. Patient survival was 99.1% at year 1, 90.5% at year 5, and 88.2% at year 10 of follow-up. Overall patient survival was 88.2% (95% confidence interval [CI]: 76.6–94.2%) after 10 years of follow-up. **b** Survival according to the type of COL3A1 pathogenic variant. Patient survival did not significantly differ between groups of pathogenic variants (Group I: 85.3% [95% CI: 69.7–93.3%] vs. Group II: 92.3% [95% CI: 56.7–98.9%] vs. Group III: 100%). Log-rank (Group I vs. Group II vs. Group III) *p* = 0.499. **c** Survival by treatment group. Survival at the end of follow-up was 93.3% for those on BB&ARB [95% CI: 79.49–97.93%] and 42.67% for those on no treatment [95% CI: 69.80–76.14%]. **d** Treated vs untreated. Survival at 5 years was 42.67% for those not on treatment [95% CI: 6.98–76.14%] and 96.53% for those on medication [95% CI: 89.35% to 98.9%]. **e** Groups I and II combining treatment types. Survival for those on BB/ARB/BB&ARB was 98.72% at 1 year, 96.89% at 5 years, and 93.95% at 10 years.

Figure 2a shows overall patient survival since first seen by the service. The survival of patients was 99.14% at 1 year, 96.10% at 3 years and 90.53% at 5 years, comparable with Frank et al. 2019, who report 99.3% survival at 1 year and 89.9% at 5 years [10]. We looked at survival for different treatment groups separately and combined (Fig. 2c–e). The results showed a significant difference in survival (log-rank test *p*-value = 0.0002) between the group of patients being treated and those not on treatment (Fig. 2d). Survival at 5 years was 96.53% for those on medication and 42.69% for those in the control group. As there were no patients with Group III variants in the group on no treatment, we also estimated survival curves excluding those in Group III but this did not affect the results (Online Supplementary Fig. 6c). This illustrates that the difference in survival between those on treatment and those not on treatment is true for those with dominant negative variants.

## Discussion

We have described our 12-year experience in a single national centre. This includes details of clinical features, events, and management of 180 molecularly proven vEDS patients, 126 of whom were available for therapeutic evaluation.

### Phenotypic features

Diagnosing vEDS early in its natural history affords the best chance of benefit from lifestyle and therapeutic interventions. We found 54% of probands had inherited the condition from a parent, showing the importance of taking a family history. Given the rarity of this condition, it is currently still most common for a diagnosis to be made following a vascular event or hollow organ rupture. Better understanding of the phenotype will help us recognise less apparent individuals who have not yet had events to consider molecular genetic testing. In addition to positive *COL3A1* molecular results, all patients in this study had detailed phenotypic assessment.

Characteristic facial features were present in the majority of our cohort and are very helpful for directing genetic testing. These features can be very subtle and, as they are not present in everyone, should not exclude further investigations if absent. Facial features characteristic of vEDS include: prominent eyes - appearing large or deep-set, a delicate midface with a thin nose and lips in comparison to other family members, and attached/lobeless ears. Thin skin, acrogeria and thinning hair resembling androgenic alopecia may be further clues to prompt consideration of the diagnosis in combination with other features.

Additional early signs identified in our cohort include: premature birth, amniotic band sequence, severe talipes, significant easy bruising, distal contractures, small joint hypermobility, pneumothorax and early onset varicose veins. Of those who were born prematurely 17/48 were paternally inherited while 15/48 were maternally inherited. Of those not born prematurely 31/90 were paternally inherited while 27/90 were maternally inherited. This is consistent with a recent study that found maternal vEDS status was not associated with preterm risk, concluding that the molecular status of the foetus alone causes the increased risk of prematurity [17].

Vascular events were 6 times more common in probands compared to relatives while bowel ruptures were 3 times more common. The comparably higher number of relatives who had previously had bowel rupture suggests this feature may be overlooked. We see the prime ages for bowel perforations being late teens and early 20s, younger than the median age of diagnosis in our cohort (29 years). It is important that spontaneous bowel perforation prompts genetic testing for vEDS in all cases.

Keratoconus and congenital hip dislocation were very rare in our cohort, despite being minor criteria on the current diagnostic criteria

### Medication

There is currently a lack of evidence regarding the beneficial use of BB and ARB in vEDS. This is an observational study not a randomised controlled clinical trial, so is not designed to specifically compare the effects of medication. It nevertheless provides important real-life data on outcomes of vEDS patients on and off these medications over a median period of almost 5 years. We acknowledge the lack of randomisation and potential for confounding issues that are limitations to analysis. Accepting these limitations, we chose to compare outcomes from patients on drug therapy with the natural comparator in our cohort, namely those who chose not to take such therapy.

The Kaplan-Meyer survival curves show a significant difference in mortality between all the treatment groups combined and those on no medication. In addition, the patients on treatment had a statistically significantly lower clinical progression score than those not on medication, suggesting a reduction in vascular events from this therapy. This was despite the treated group having a higher number of patients with vascular events at baseline (36%) compared to those on no treatment (20%). All patients in the analysis were seen by the same clinical staff in the same service, were given the same lifestyle and management advice and remained under follow-up at the end of the study period. The majority of our cohort were on a combination of losartan and bisoprolol. It is not known whether there may be significant clinical differences in the vascular effects of different ARB or BB drugs in this situation, or whether any benefit is a class effect.

Survival in the treated group (ARB&BB) was 93.3% over a median of 4.67 years, comparable with the previous study using celiprolol [10] which showed survival of 85% over a median of 5.1 years for those taking celiprolol 400 mg/day. We had only a small number of patients on celiprolol, so we cannot draw conclusions regarding celiprolol efficacy from this group.

We have found both BB and ARB to be well tolerated. Adverse effects are an important consideration, given that patients are taking medication in view of their perceived risk, rather than to alleviate symptoms. Adverse effects have generally been minor, in which case the dosage has been kept lower. We have aimed to titrate to the maximum tolerated dose within the usual dose range. Asymptomatic low blood pressure has not been regarded as an indication to discontinue therapy, although in some cases this stops further up-titration.

### Management

Genetic counselling following diagnosis can help patients and families to understand and adapt to the diagnosis as well as enabling cascade testing for other family members. Lifestyle advice is important in vEDS, including discussion about activities that may increase the risks of vascular or hollow organ rupture. Regular, low-intensity exercise should be maintained for health and psychological wellbeing.

Our current imaging surveillance protocol is annual MRA of the whole of the thoracic and abdominal aorta, cervical vessels including the circle of Willis, and distally to the pelvis/upper legs. Any evolving changes are assessed with specific imaging at shorter intervals as appropriate. CT angiography is used sparingly and focused on specific arterial territories where there are MRA imaging abnormalities.

Given the risk for bowel perforation, procedures such as colonoscopy should be considered carefully and avoided where possible. Following bowel perforation, the suggested treatment option to reduce reperforation is total colectomy and ileostomy [7]. As surgery is usually performed in an emergency setting, where the vEDS diagnosis is not known, this is seldom done. We found the most common site for bowel perforations to be the large intestine, specifically the sigmoid colon, as with previous studies [4, 7]. Small intestinal perforation occurs but is unusual. The number of patients who had further bowel ruptures supports the view that colostomies should not be reversed for vEDS patients due to risk for re-perforation.

Patients with vEDS often have difficulties when they need to access emergency care. The rarity of the condition results in a lack of awareness in Emergency Departments. The propensity to sudden, severe, and life-threatening complications, and also of the serious difficulties that can occur when surgical intervention is carried out need to be considered. To support patient self-advocacy, we have provided an ‘emergency information for medical professionals’ card for people with vEDS to carry. This ensures that the correct medical management can take place. In the UK this is included as an alert on electronic patient records (Online Supplementary Fig. 4). Surgical risks are higher for those with vEDS and conservative management options should be considered before surgery. Where surgical management is essential, outcomes are better in an elective setting [8].

Pregnancy is known to carry significant risks for women with vEDS with vascular events, uterine rupture, 4th-degree tears, hemorrhage and death reported [6]. We had no patients with uterine rupture but saw a similar rate of maternal death and life-threatening vascular events as reported in previous studies [6], all in patients who had their pregnancy prior to diagnosis. There are currently no international guidelines for managing pregnancy in women with vEDS. We have been involved in managing pregnancies in women diagnosed with vEDS and these have all been carefully coordinated with specialist obstetric teams in tertiary care. We presented this experience with a poster at the International Scientific Symposium on EDS & HSD in 2022 (Online Supplementary Fig. 5) [18].

## Conclusion

Our study provides detailed phenotypic information, long-term clinical and imaging follow up and the effects of drug therapies in 126 molecularly proven vEDS cases.

The phenotypic spectrum is broader than previously recognised. There are a number of early clinical clues to prompt genetic testing before vascular/hollow organ rupture. These include significant easy bruising, severe talipes, premature birth, amniotic band sequence, distal contractures, pneumothorax and early onset varicose veins. Clinical events listed as the 2017 major criteria should of course initiate genetic investigation, but bowel rupture may still be under recognised as a reason for referral. Distal contractures and amniotic band sequence do not appear on the current diagnostic criteria despite being more frequent in our cohort than keratoconus and congenital hip dysplasia, both currently minor criteria [2].

We have highlighted the importance of lifestyle measures and the key role of MDT discussion for management around pregnancy and other surgical interventions. The tissue fragility risks cause both a higher incidence of sudden, severe and life-threatening complications, as well as creating additional challenges for medical intervention.

The observation of improved clinical outcomes in patients on ARB/BB therapy compared with those not on treatment has potentially important clinical consequences and is worthy of further research. In the absence of randomised controlled trial results, it supports the use of these agents in this very high-risk group, until such data becomes available.

We highlight the role of the specialist service in managing this rare condition and demonstrate the need for early diagnosis of vEDS. With early diagnosis, appropriate medical management, lifestyle advice, and medication we can improve survival and quality of life for people with vEDS.

## Supplementary information

Online Supplement

## Data Availability

The datasets generated during and/or analysed during the current study are available in the Global Variome shared LOVD repository, [https://gbr01.safelinks.protection.outlook.com/?url=https%3A%2F%2Fdatabases.lovd.nl%2Fshared%2Findividuals%3Fsearch_created_by%3D04452&data=05%7C01%7Cjessica.bowen4%40nhs.net%7Cfe0296e8b9894528e42208db0e81b57e%7C37c354b285b047f5b22207b48d774ee3%7C0%7C0%7C638119724054592498%7CUnknown%7CTWFpbGZsb3d8eyJWIjoiMC4wLjAwMDAiLCJQIjoiV2luMzIiLCJBTiI6Ik1haWwiLCJXVCI6Mn0%3D%7C3000%7C%7C%7C&sdata=i7CoqJOUqJUwC2JcArocFF0dCr1W1z1%2FV6FWdVAWMcE%3D&reserved=0].

## References

[1] Shalhub S, Byers PH, Hicks KL, Coleman DM, Davis FM, De Caridi G (2020). A multi-institutional experience in vascular Ehlers-Danlos syndrome diagnosis. J Vasc Surg.

[2] Malfait F, Francomano C, Byers P, Belmont J, Berglund B, Black J (2017). The 2017 International Classification of the Ehlers–Danlos Syndromes. Am J Med Genet C.

[3] Pepin M, Schwarze U, Superti-Furga A, Byers PH (2000). Clinical and genetic features of Ehlers–Danlos syndrome type IV, the vascular type. N Engl J Med.

[4] Pepin MG, Schwarze U, Rice KM, Liu M, Leistritz D, Byers PH (2014). Survival is affected by mutation type and molecular mechanism in vascular Ehlers–Danlos syndrome (EDS type IV). Genet Med.

[5] Frank M, Albuisson J, Ranque B, Golmard L, Mazzella J, Bal-Theoleyre L (2015). The type of variants at the COL3A1 gene associates with the phenotype and severity of vascular Ehlers–Danlos syndrome. Eur J Hum Genet.

[6] Murray ML, Pepin M, Peterson S, Byers PH (2014). Pregnancy-related deaths and complications in women with vascular Ehlers–Danlos syndrome. Genet Med.

[7] Frank M, Adham S, Zinzindohoué F, Jeunemaitre X (2019). Natural history of gastrointestinal manifestations in vascular Ehlers–Danlos syndrome: A 17-year retrospective review. J Gastroenterol Hepatol.

[8] Shalhub S, Black JH, Cecchi AC, Xu Z, Griswold BF, Safi HJ (2014). Molecular diagnosis in vascular Ehlers-Danlos syndrome predicts pattern of arterial involvement and outcomes. J Vasc Surg.

[9] Shalhub S, Byers PH, Hicks KL, Charlton-Ouw K, Zarkowsky D, Coleman DM (2019). A multi-institutional experience in the aortic and arterial pathology in individuals with genetically confirmed vascular Ehlers-Danlos syndrome. J Vasc Surg.

[10] Frank M, Adham S, Seigle S, Legrand A, Mirault T, Henneton P (2019). Vascular Ehlers-Danlos syndrome: Long-term observational study. J Am Coll Cardiol.

[11] Shalhub S, Neptune E, Sanchez DE, Dua A, Arellano N, Mcdonnell NB (2019). Spontaneous pneumothorax and hemothorax frequently precede the arterial and intestinal complications of vascular Ehlers–Danlos syndrome. Am J Med Genet A..

[12] Singh MN, Lacro RV (2016). Recent clinical drug trials evidence in Marfan syndrome and clinical implications. Can J Cardiol.

[13] Ong KT, Perdu J, De Backer J, Bozec E, Collignon P, Emmerich J (2010). Effect of celiprolol on prevention of cardiovascular events in vascular Ehlers-Danlos syndrome: A prospective randomised, open, blinded-endpoints trial. Lancet..

[14] Ellard S, Baple EL, Berry I, Forrester N, Turnbull C, Owens MM, et al. ACGS Best Practice Guidelines for Variant Classification. 2019.

[15] Statacorp. 2021. Stata Statistical Software: Release 17. College Station, Tx: Statacorp LLC.

[16] Schirwani S, Van Dijk FS, Cauldwell M, Harrison RS, Kraus A, Brennan P (2022). Amniotic band sequence in vascular Ehlers-Danlos syndrome (EDS): Experience of the EDS National Diagnostic Services in the UK. Eur J Med Genet.

[17] Stephens SB, Russo M, Shalhub S, Beecroft T, Weigand J, Milewicz DM (2022). Evaluating perinatal and neonatal outcomes among children with vascular Ehlers-Danlos syndrome. Genet Med.

[18] Bowen J Abstract 38: Vascular EDS and Pregnancy, Poster Presentation. Proceedings of the 2022 International Scientific Symposium on EDS & HSD. 2022, Sept 14–17; Rome.

