# Supplementary material for: Diagnosis and management of vascular Ehlers-Danlos syndrome: Experience of the UK national diagnostic service, Sheffield
Source: Eur J Hum Genet. 2023 Mar 29;31(7):749–60. doi: 10.1038/s41431-023-01343-7 (PMC10326011; doi:10.1038/s41431-023-01343-7)
Supplement: Supplementary file 1 — Online Supplement [file 41431_2023_1343_MOESM1_ESM.pdf]

# Supplementary Material

Online Table 1: molecular results and clinical data for full vEDS cohort. PM=post mortem. *Variants submitted to the Global Variome shared LOVD.

| Sex | Age at diagnosis | Proband/ Relative | Molecular result* | Variant Group | Inheritance | vEDS related events | Pregnancy before/ after diagnosis | Deceased | In statistical analysis cohort |
|---|---|---|---|---|---|---|---|---|---|
| female | 22 | relative | c.539G>A p.(Gly180Asp) | 1 | paternally inherited | haemoptysis age 22 | 0 | no | Yes |
| male | 30 | proband | c.539G>A p.(Gly180Asp) | 1 | unknown | arterial events | | abdominal haemorrhage age 31 | No |
| female | 27 | proband | c.547G>A p.(Gly183Ser) | 1 | de novo | arterial events bowel: sigmoid colon age 9, small bowel age 21 | 0 | renal infarction, infracolic haematoma age 28 | Yes |
| female | 28 | proband | c.584G>T p.(Gly195Val) | 1 | paternally inherited | arterial events bowel: large bowel age 29, small bowel age 29 | 0 | no | No |
| male | 29 | proband | c.593G>T p.(Gly198Val) | 1 | unknown | arterial events multiple pneumothorax age 26 | | no | Yes |
| female | 28 | proband | c.610G>C p.(Gly204Arg) | 1 | unknown | arterial events | 1 before | no | Yes |
| male | 10 months | relative | c.610G>C p.(Gly204Arg) | 1 | maternally inherited | pneumothorax age 1 day | | no | No |
| male | 7 | proband | c.656G>A p.(Gly219Asp) | 1 | paternally inherited | no | | no | Yes |
| female | 18 | proband | c.664G>C p.(Gly222Arg) | 1 | maternally inherited | no | 0 | no | Yes |
| female | 27 | Relative | c.755G>T p.(Gly252Val) | 1 | paternally inherited | arterial events | 0 | no | Yes |
| female | 30 | proband | c.755G>T p.(Gly252Val) | 1 | unknown | arterial events | 0 | no | Yes |
| male | 33 | proband | c.755G>T p.(Gly252Val) | 1 | paternally inherited | arterial events | | no | Yes |
| male | 59 | Relative | c.755G>T p.(Gly252Val) | 1 | maternally inherited | arterial events | | no | No |
| male | 13 | relative | c.755G>T p.(Gly252Val) | 1 | paternally inherited | no | | no | Yes |
| male | 16 | proband | c.755G>T p.(Gly252Val) | 1 | paternally inherited | bowel rupture: sigmoid colon age 16 | | no | Yes |
| male | PM | relative | c.755G>T p.(Gly252Val) | 1 | unknown | arterial events pneumothorax multiple | | arterial dissection age 34 | No |

| Sex | Age at diagnosis | Proband/ Relative | Molecular result* | Variant Group | Inheritance | vEDS related events | Pregnancy before/ after diagnosis | Deceased | In statistical analysis cohort |
|---|---|---|---|---|---|---|---|---|---|
| female | 38 | relative | c.845G>A p.(Gly282Glu) | 1 | maternally inherited | no | 2 before & after | no | Yes |
| female | 56 | proband | c.845G>A p.(Gly282Glu) | 1 | de novo | arterial events | 3 before | no | Yes |
| male | 2 | relative | c.845G>A p.(Gly282Glu) | 1 | maternally inherited | no | | no | No |
| female | 29 | proband | c.899G>A p.(Gly300Asp) | 1 | paternally inherited | arterial events | 0 | no | Yes |
| female | 18 | proband | c.970G>A p.(Gly324Ser) | 1 | unknown | arterial events | 1 after | no | Yes |
| male | 33 | proband | c.970G>A p.(Gly324Ser) | 1 | paternally inherited | arterial events | | no | Yes |
| female | 23 | relative | c.998G>A p.(Gly333Asp) | 1 | paternally inherited | no | 0 | no | Yes |
| male | 16 | relative | c.998G>A p.(Gly333Asp) | 1 | paternally inherited | pneumothorax x2 age 14 & 15 | | no | Yes |
| male | 18 | relative | c.998G>A p.(Gly333Asp) | 1 | paternally inherited | arterial events | | aortic dissection age 19 | Yes |
| male | PM | proband | c.998G>A p.(Gly333Asp) | 1 | paternally inherited | arterial events | | aortic dissection age 17 | No |
| female | 18 | proband | c.1007G>A p.(Gly336Asp) | 1 | paternally inherited | bowel rupture: large bowel age 12, small bowel age 13 | 0 | no | Yes |
| male | 32 | relative | c.1007G>A p.(Gly336Asp) | 1 | maternally inherited | arterial events pneumothorax, bowel perforation age 13, 16, 30 | | dissecting thoracic aneurysm age 44 | No |
| female | 8 months | relative | c.1241G>A p.(Gly414Asp) | 1 | maternally inherited | no | | no | No |
| female | PM | proband | c.1241G>A p.(Gly414Asp) | 1 | unknown | arterial events | 1 before | aortic dissection 7 days post c-section age 33 | No |
| male | 7 | relative | c.1258G>A p.(Gly420Ser) | 1 | paternally inherited | no | | no | No |
| male | 20 | relative | c.1258G>A p.(Gly420Ser) | 1 | paternally inherited | no | | no | Yes |
| male | 48 | proband | c.1258G>A p.(Gly420Ser) | 1 | paternally inherited | arterial events pneumothorax age 38 | | no | Yes |
| male | PM | relative | c.1258G>A p.(Gly420Ser) | 1 | paternally inherited | arterial events pneumothorax x2 | | aortic dissection age 43 | No |

| Sex | Age at diagnosis | Proband/ Relative | Molecular result* | Variant Group | Inheritance | vEDS related events | Pregnancy before/ after diagnosis | Deceased | In statistical analysis cohort |
|---|---|---|---|---|---|---|---|---|---|
| male | 31 | Proband | c.1295G>T p.(Gly432Val) | 1 | de novo | arterial events bowel age 22 | | no | Yes |
| female | 26 | proband | c.1330G>A p.(Gly444Arg) | 1 | paternally inherited | arterial events bowel age 25 | 2 before | no | Yes |
| male | 17 | relative | c.1330G>A p.(Gly444Arg) | 1 | maternally inherited | bowel rupture: sigmoid colon age 22 | | no | Yes |
| female | 47 | proband | c.1502G>A p.(Gly501Glu) | 1 | unknown | arterial events | 2 before | vessel rupture in abdomen age 51 | Yes |
| female | 24 | relative | c.1555G>A p.(Gly519Arg) | 1 | maternally inherited | bowel rupture: sigmoid colon age 23 | 2 before | no | Yes |
| female | 30 | relative | c.1555G>A p.(Gly519Arg) | 1 | maternally inherited | bowel rupture: sigmoid colon age 29 | 2 before | no | Yes |
| female | 51 | proband | c.1555G>A p.(Gly519Arg) | 1 | unknown | bowel rupture: sigmoid colon age 45 | 2 before | no | Yes |
| male | 19 | proband | c.1618G>A p.(Gly540Arg) | 1 | maternally inherited | arterial events | | no | Yes |
| female | 46 | proband | c.1700G>A p.(Gly567Glu) | 1 | paternally inherited | arterial events splenectomy age 13, bowel: colon age 13 | 0 | no | Yes |
| female | 26 | relative | c.1744G>A p.(Gly582Ser) | 1 | maternally inherited | no | 3 before | no | Yes |
| female | 52 | relative | c.1744G>A p.(Gly582Ser) | 1 | maternally inherited | arterial events | 2 before | pancreatic cancer age 55 | Yes |
| male | 5 | relative | c.1744G>A p.(Gly582Ser) | 1 | maternally inherited | no | | no | No |
| male | 28 | proband | c.1763G>A p.(Gly588Asp) | 1 | unknown | haemoptysis & haemothorax age 24 | | no | Yes |
| male | 20 | proband | c.1915G>A p.(Gly639Arg) | 1 | de novo | no | | no | Yes |
| female | 6 | Relative | c.1988G>A p.(Gly663Asp) | 1 | paternally inherited | no | | no | No |
| male | 42 | proband | c.1988G>A p.(Gly663Asp) | 1 | unknown | arterial events | | no | Yes |
| male | 33 | proband | c.1996G>A p.(Gly666Ser) | 1 | paternally inherited | arterial events | | no | Yes |
| male | 39 | proband | c.2032G>C p.(Gly678Arg) | 1 | maternally inherited | arterial events | | no | Yes |
| female | 16 | relative | c.2068G>A p.(Gly690Arg) | 1 | maternally inherited | no | 0 | no | Yes |
| female | 46 | relative | c.2068G>A p.(Gly690Arg) | 1 | paternally inherited | no | 5 before | no | Yes |

| Sex | Age at diagnosis | Proband/ Relative | Molecular result* | Variant Group | Inheritance | vEDS related events | Pregnancy before/ after diagnosis | Deceased | In statistical analysis cohort |
|---|---|---|---|---|---|---|---|---|---|
| female | 52 | relative | c.2068G>A p.(Gly690Arg) | 1 | paternally inherited | arterial events | 3 before | no | Yes |
| female | 55 | relative | c.2068G>A p.(Gly690Arg) | 1 | paternally inherited | arterial events | 2 before | no | Yes |
| male | 25 | relative | c.2068G>A p.(Gly690Arg) | 1 | maternally inherited | no | | no | Yes |
| male | PM | proband | c.2068G>A p.(Gly690Arg) | 1 | unknown | arterial events | | dissecting thoracic aortic aneurysm age 57 | No |
| female | PM | relative | c.2105G>T p.(Gly702Val) | 1 | de novo | arterial events | 1 before | dissected iliac artery in childbirth age 30 | No |
| male | 12 | proband | c.2105G>T p.(Gly702Val) | 1 | maternally inherited | arterial events | | no | Yes |
| female | 53 | proband | c.2123G>C p.(Gly708Ala) | 1 | unknown | arterial events | 0 | no | Yes |
| female | 38 | proband | c.2177G>T p.(Gly726Val) | 1 | de novo | arterial events | 2 before | no | Yes |
| female | 36 | proband | c.2177G>T p.(Gly726Val) | 1 | unknown | arterial events bowel rupture age 36 | 1 before | no | No |
| male | 8 | relative | c.2177G>T p.(Gly726Val) | 1 | maternally inherited | no | | no | Yes |
| female | 43 | proband | c.2285G>T p.(Gly762Val) | 1 | unknown | arterial events bowel: descending colon age 19 & large bowel age 20 | 0 | no | Yes |
| female | 49 | Relative | c.2329G>C p.(Gly777Arg) | 1 | unknown | no | 1 before | no | Yes |
| female | 40 | relative | c.2329G>C p.(Gly777Arg) | 1 | paternally inherited | no | 3 before | no | Yes |
| female | 41 | relative | c.2329G>C p.(Gly777Arg) | 1 | paternally inherited | arterial events | 2 before | no | Yes |
| female | 34 | relative | c.2329G>C p.(Gly777Arg) | 1 | paternally inherited | no | 4 before | no | Yes |
| female | 36 | relative | c.2329G>C p.(Gly777Arg) | 1 | paternally inherited | no | 4 before | no | Yes |
| female | 7 | relative | c.2329G>C p.(Gly777Arg) | 1 | maternally inherited | no | | no | No |
| female | 13 | relative | c.2329G>C p.(Gly777Arg) | 1 | maternally inherited | no | | no | Yes |
| female | 22 | relative | c.2329G>C p.(Gly777Arg) | 1 | maternally inherited | no | 0 | no | Yes |
| female | 1 | relative | c.2329G>C p.(Gly777Arg) | 1 | maternally inherited | no | | no | No |

| Sex | Age at diagnosis | Proband/ Relative | Molecular result* | Variant Group | Inheritance | vEDS related events | Pregnancy before/ after diagnosis | Deceased | In statistical analysis cohort |
|---|---|---|---|---|---|---|---|---|---|
| male | 22 | proband | c.2329G>C p.(Gly777Arg) | 1 | maternally inherited | arterial events pneumothorax age 25 | | no | Yes |
| male | 14 | relative | c.2329G>C p.(Gly777Arg) | 1 | maternally inherited | no | | no | Yes |
| male | 10 | relative | c.2329G>C p.(Gly777Arg) | 1 | maternally inherited | no | | no | No |
| male | 62 | proband | c.2329G>C p.(Gly777Arg) | 1 | inherited - unsure which parent | arterial events | | no | Yes |
| female | 3 | relative | c.2339G>A p.(Gly780Asp) | 1 | maternally inherited | no | | no | No |
| female | 29 | relative | c.2339G>A p.(Gly780Asp) | 1 | maternally inherited | no | 2 before | no | Yes |
| female | 19 | relative | c.2356G>A p.(Gly786Arg) | 1 | maternally inherited | no | 0 | no | Yes |
| female | 52 | proband | c.2356G>A p.(Gly786Arg) | 1 | paternally inherited | arterial events (diverticulae perforation age 54) | 4 before | no | Yes |
| female | 25 | relative | c.2356G>A p.(Gly786Arg) | 1 | maternally inherited | no | 0 | no | Yes |
| male | 4 | relative | c.2356G>A p.(Gly786Arg) | 1 | paternally inherited | no | | no | No |
| male | 9 | relative | c.2356G>A p.(Gly786Arg) | 1 | paternally inherited | no | | no | No |
| male | 40 | relative | c.2356G>A p.(Gly786Arg) | 1 | maternally inherited | no | | no | Yes |
| female | 50 | relative | c.2401G>C p.(Gly801Arg) | 1 | maternally inherited | bowel rupture age 31 (surgical bowel perforation age 40) | 1 before | no | Yes |
| female | 74 | relative | c.2401G>C p.(Gly801Arg) | 1 | unknown | no | 1 before | no | No |
| male | 22 | proband | c.2401G>C p.(Gly801Arg) | 1 | maternally inherited | bowel rupture age 16 | | no | Yes |
| female | 2 | proband | c.2483G>A p.(Gly828Glu) | 1 | paternally inherited | no | | no | No |
| male | 42 | proband | c.2491G>C p.(Gly831Arg) | 1 | paternally inherited | haemoptysis age 37 | | no | Yes |
| female | 11 | Relative | c.2554G>T p.(Gly852Cys) | 1 | paternally inherited | no | 0 | no | Yes |
| male | 37 | proband | c.2554G>T p.(Gly852Cys) | 1 | paternally inherited | arterial events | | no | Yes |
| male | PM | proband | c.2798G>A p.(Gly933Glu) | 1 | unknown | arterial events | | ruptured vena cava age 15 | No |
| female | 22 | Relative | c.2834G>A p.(Gly945Asp) | 1 | maternally inherited | no | 0 | no | Yes |
| female | 24 | Relative | c.2834G>A p.(Gly945Asp) | 1 | maternally inherited | no | 0 | no | Yes |

| Sex | Age at diagnosis | Proband/ Relative | Molecular result* | Variant Group | Inheritance | vEDS related events | Pregnancy before/ after diagnosis | Deceased | In statistical analysis cohort |
|---|---|---|---|---|---|---|---|---|---|
| female | PM | Relative | c.2834G>A p.(Gly945Asp) | 1 | unknown | arterial events | 4 before | died during heart valve surgery age 39 | No |
| male | PM | proband | c.2834G>A p.(Gly945Asp) | 1 | maternally inherited | arterial events | | coronary artery rupture age 24 | No |
| female | 62 | relative | c.2851G>A p.Gly951Arg | 1 | unknown | arterial events | 3 before | no | Yes |
| male | 2 | relative | c.2851G>A p.Gly951Arg | 1 | paternally inherited | no | | no | No |
| male | 31 | relative | c.2851G>A p.Gly951Arg | 1 | maternally inherited | no | | no | Yes |
| male | PM | proband | c.2851G>A p.Gly951Arg | 1 | maternally inherited | arterial events | | ruptured renal artery age 34 | No |
| male | 23 | proband | c.2923G>A p.(Gly975Ser) | 1 | maternally inherited | arterial events | | severe retroperitoneal bleeding age 24 | Yes |
| male | 12 | proband | c.2924G>T p.(Gly975Val) | 1 | de novo | arterial events | | aortic dissection age 20 | Yes |
| female | 46 | proband | c.2951G>T p.(Gly984Val) | 1 | unknown | arterial events | 2 before | no | Yes |
| female | 38 | proband | c.3004G>A p.(Gly1002Ser) | 1 | paternally inherited | arterial events bowel rupture age 25 | 4 before | no | Yes |
| female | 28 | relative | c.3004G>A p.(Gly1002Ser) | 1 | paternally inherited | no | 0 | no | Yes |
| male | PM | relative | c.3004G>A p.(Gly1002Ser) | 1 | unknown | arterial events | | aortic dissection age 64 | No |
| female | 20 | Relative | c.3067G>C p.(Gly1023Arg) | 1 | maternally inherited | haemoptysis age 23 | 2 before | no | Yes |
| female | 51 | Relative | c.3067G>C p.(Gly1023Arg) | 1 | paternally inherited | arterial events | 1 before | no | Yes |
| female | 46 | proband | c.3067G>C p.(Gly1023Arg) | 1 | paternally inherited | arterial events | 2 before | no | Yes |
| male | PM | relative | c.3067G>C p.(Gly1023Arg) | 1 | maternally inherited | no | | seizures secondary to frontal lobe infarction age 19 | No |
| male | 4 months | relative | c.3067G>C p.(Gly1023Arg) | 1 | maternally inherited | no | | no | No |

| Sex | Age at diagnosis | Proband/ Relative | Molecular result* | Variant Group | Inheritance | vEDS related events | Pregnancy before/ after diagnosis | Deceased | In statistical analysis cohort |
|---|---|---|---|---|---|---|---|---|---|
| female | 26 | proband | c.3104G>T p.(Gly1035Val) | 1 | inherited - unsure which parent | arterial events bowel rupture age 15 | 1 after | dissection of thoracic aorta age 31 | Yes |
| male | 1 | Relative | c.3104G>T p.(Gly1035Val) | 1 | paternally inherited | no | | no | No |
| male | 25 | Relative | c.3104G>T p.(Gly1035Val) | 1 | inherited - unsure which parent | arterial events | | ruptured thoracic aorta age 29 | Yes |
| female | 59 | Relative | c.3149G>A p.(Gly1050Asp) | 1 | unknown | arterial events | 3 before | no | Yes |
| female | PM | proband | c.3149G>A p.(Gly1050Asp) | 1 | maternally inherited | arterial events | 1 before | aortic dissection age 34 | No |
| male | 2 | Relative | c.3149G>A p.(Gly1050Asp) | 1 | maternally inherited | no | | no | No |
| female | 43 | proband | c.3212G>T p.(Gly1071Val) | 1 | unknown | arterial events haemoptysis - multiple episodes age 44 | 0 | no | Yes |
| male | 2 | proband | c.3284G>A p.(Gly1095Asp) | 1 | de novo | no | | no | No |
| female | 67 | relative | c.3391G>A p.(Gly1131Ser) | 1 | inherited - unsure which parent | no | 1 before | no | Yes |
| female | 13 | relative | c.3391G>A p.(Gly1131Ser) | 1 | paternally inherited | no | | no | Yes |
| female | 63 | relative | c.3391G>A p.(Gly1131Ser) | 1 | inherited - unsure which parent | no | 4 before | no | Yes |
| male | 45 | relative | c.3391G>A p.(Gly1131Ser) | 1 | maternally inherited | no | | no | Yes |
| male | 64 | relative | c.3391G>A p.(Gly1131Ser) | 1 | inherited - unsure which parent | no | | no | Yes |
| male | 11 | proband | c.3500G>A p.(Gly1167Asp) | 1 | paternally inherited | no | | no | No |
| male | 38 | relative | c.3500G>A p.(Gly1167Asp) | 1 | unknown | no | | no | Yes |
| male | 6 | proband | c.3554G>T p.(Gly1185Val) | 1 | de novo | pneumothorax age 12 weeks | | no | Yes |
| female | 27 | relative | c.537_554del p.(Pro181_Gly186del) | 2 | unknown | no | 3 before | no | Yes |
| male | 11 | proband | c.582+1G>A p.(?) | 2 | maternally inherited | no | | no | No |

| Sex | Age at diagnosis | Proband/ Relative | Molecular result* | Variant Group | Inheritance | vEDS related events | Pregnancy before/ after diagnosis | Deceased | In statistical analysis cohort |
|---|---|---|---|---|---|---|---|---|---|
| female | PM | relative | c.690+1G>A p.(?) | 2 | unknown | arterial events spleen rupture age 27 | 2 before | abdominal haemorrhage age 30 | No |
| male | 8 | relative | c.690+1G>A p.(?) | 2 | paternally inherited | no | | no | No |
| male | 12 | relative | c.690+1G>A p.(?) | 2 | paternally inherited | no | | no | No |
| male | 33 | proband | c.690+1G>A p.(?) | 2 | maternally inherited | arterial events | | no | Yes |
| male | PM | relative | c.690+1G>A p.(?) | 2 | maternally inherited | arterial events spleen rupture age 12, bowel: sigmoid colon age 35 | | ruptured right renal artery aneurysm age 38 | No |
| female | 53 | proband | c.952-1G>T p.(?) | 2 | paternally inherited | no | 2 before | no | Yes |
| female | 28 | proband | c.996+5G>A p.(?) | 2 | maternally inherited | no | 0 | no | Yes |
| female | 47 | proband | c.996+5G>A p.(?) | 2 | de novo | arterial events | 3 before | no | Yes |
| female | 40 | proband | c.1347+1G>A p.(?) | 2 | maternally inherited | arterial events | 1 before | no | Yes |
| female | 37 | proband | c.1662+1G>A p.(?) | 2 | unknown | arterial events bowel rupture age 22 | 1 diagnosis made during pregnancy | no | Yes |
| female | 9 | proband | c.1662+1G>C p.(?) | 2 | paternally inherited | no | 0 | no | Yes |
| female | 3 | proband | c.1662+1G>A p.(?) | 2 | de novo | no | | no | No |
| female | 11 | proband | c.1662+1G>A p.(?) | 2 | unknown | bowel rupture: rectum age 11, colon age 12 | 0 | no | Yes |
| male | 20 | proband | c.1662+1G>A p.(?) | 2 | unknown | pneumothorax age 22 | | no | Yes |
| female | 30 | proband | c.1761+5G>A p.(?) | 2 | de novo | arterial events | 0 | no | Yes |
| female | 3 | relative | c.1761+1G>A p.(?) | 2 | maternally inherited | no | | no | No |
| female | 37 | proband | c.1761+1G>A p.(?) | 2 | maternally inherited | arterial events | 1 before | no | Yes |
| female | 15 | proband | c.2230-3_2255del p.(?) | 2 | de novo | arterial events | 0 | aortic dissection age 19 | Yes |
| male | 57 | proband | c.2392-2A>G p.(?) | 2 | inherited - unsure which parent | arterial events | | no | Yes |
| male | 5 | proband | c.2445+3_2445+6del p.(?) | 2 | de novo | no | | no | No |

| Sex | Age at diagnosis | Proband/ Relative | Molecular result* | Variant Group | Inheritance | vEDS related events | Pregnancy before/ after diagnosis | Deceased | In statistical analysis cohort |
|---|---|---|---|---|---|---|---|---|---|
| male | 16 | proband | c.3039+3G>C p.(?) | 2 | de novo | bowel rupture age 16 | | no | Yes |
| female | 22 | relative | c.3201+1G>C p.(?) | 2 | unknown | no | 1 before | no | Yes |
| male | prenatal | proband | c.3201+1G>C p.(?) | 2 | maternally inherited | no | | no | No |
| female | 50 | relative | c.3213_3221dup p.(Pro1072_Gly1074dup) | 2 | unknown | arterial events | 2 before | no | Yes |
| female | 38 | proband | c.3525+1G>A p.(?) | 2 | unknown | no | 0 | no | Yes |
| male | 18 | proband | c.3525+2T>C p.(?) | 2 | unknown | no | | no | Yes |
| male | 15 | proband | c.3823+1G>A p.(?) | 2 | de novo | no | | no | Yes |
| female | 4 | proband | c.20022+2T>C p.(?) | 2 | de novo | no | 0 | no | Yes |
| female | 17 | Relative | c.382C>T p.(Gln128*) | 3 | paternally inherited | no | 0 | no | Yes |
| male | 51 | proband | c.382C>T p.(Gln128*) | 3 | inherited - unsure which parent | arterial events | | no | Yes |
| female | 73 | Relative | c.592G>T p.(Gly198*) | 3 | unknown | no | 2 before | lung cancer age 76 | Yes |
| male | 38 | proband | c.592G>T p.(Gly198*) | 3 | maternally inherited | arterial events | | no | Yes |
| male | 6 | relative | c.592G>T p.(Gly198*) | 3 | paternally inherited | no | | no | No |
| male | 47 | proband | c.1714C>T p.(Arg572*) | 3 | paternally inherited | arterial events | | no | Yes |
| female | 44 | proband | c.1979delG p.(Gly660Valfs*131) | 3 | maternally inherited | no | 2 before | no | Yes |
| male | 22 | relative | c.1979delG p.(Gly660Valfs*131) | 3 | maternally inherited | no | | no | Yes |
| female | 27 | proband | c.2409_2422del14 p.(Gly804Cysfs*12) | 3 | inherited - unsure which parent | arterial events | 1 after | no | Yes |
| male | 28 | Relative | c.2409_2422del14 p.(Gly804Cysfs*12) | 3 | inherited - unsure which parent | arterial events Spleen rupture age 22 | | no | Yes |
| female | 20 | relative | c.2797G>T p.(Gly933*) | 3 | paternally inherited | no | 0 | no | Yes |
| male | 17 | proband | c.2797G>T p.(Gly933*) | 3 | paternally inherited | arterial events | | no | Yes |
| male | PM | relative | c.2797G>T p.(Gly933*) | 3 | maternally inherited | arterial events | | aortic dissection age 44 | No |

| Sex | Age at diagnosis | Proband/ Relative | Molecular result* | Variant Group | Inheritance | vEDS related events | Pregnancy before/ after diagnosis | Deceased | In statistical analysis cohort |
|---|---|---|---|---|---|---|---|---|---|
| female | 52 | proband | c.4138delG p.(Asp1380Ilefs*7) | 3 | unknown | bowel rupture: sigmoid colon age 46 | 4 before | no | Yes |
| male | 17 | Relative | c.4294C>T p.(Arg1432*) | 3 | paternally inherited | no | | no | Yes |
| male | PM | proband | c.4294C>T p.(Arg1432*) | 3 | maternally inherited | arterial events | | dissecting aortic aneurysm age 55 | No |
| female | 22 | proband | c.4400A>T p.(Leu1467*) | 3 | de novo | no | 1 after | no | Yes |
| female | 8 | proband | 2q deletion including *COL3A1* | 3 | de novo | no | | no | No |
| female | prenatal | proband | 2q deletion including *COL3A1* | 3 | de novo | no | | no | No |
| female | 6 | proband | 2q deletion including *COL3A1* | 3 | de novo | no | | no | No |
| female | 1 | proband | 2q deletion including *COL3A1* | 3 | de novo | no | | no | No |

Online Table 2: Clinical Arterial score (CA score)

Definition of the clinical arterial score and illustrations of patient staging from Frank et al., 2019 [10].

- 0 point: no clinical arterial event
- 1 point: a silent dissection; an aneurysm; a false aneurysm
- 2 points: a symptomatic dissection
- 5 points: an arterio-venous fistula; a lower or upper limb arterial rupture
- 10 points: a carotid-cavernous fistula; an aortic dissection and/or rupture; an abdominal artery rupture; an intrathoracic artery rupture

Points were assigned according to expected clinical consequences of each arterial type of lesion, the highest points being assigned to life-threatening arterial accidents. The arterial score can only increase throughout follow-up and boundaries were chosen to allow easy group changes, particularly for life-threatening accidents.

| | |
|---|---|
| **Group 1 (very low, score 0-1 point)** | • No clinical arterial event (0 point)<br>• A silent dissection (1 point)<br>• An aneurysm (1 point)<br>• A false aneurysm (1 point) |
| **Group 2 (low, score 2-8 points)** | • A symptomatic dissection (2 points)<br>• A symptomatic dissection (2 points) and an aneurysm (1 point) (=3 points)<br>• An arterio-venous fistula (5 points) and a symptomatic dissection (2 points) (=7 points)<br>• A lower or upper limb arterial rupture (5 points) and a symptomatic dissection (2 points) and a silent dissection (1 point) (=8 points) |
| **Group 3 (medium, score 9-19 points)** | • An arterio-venous fistula (5 points), a symptomatic dissection (2 points) and 2 silent dissections (2 points) (=9 points)<br>• A carotid-cavernous fistula (10 points)<br>• An aortic rupture (10 points) and a lower or upper limb arterial rupture (5 points) (=15 points)<br>• An intracranial artery rupture (10 points), an arteriovenous fistula (5 points), and 2 symptomatic dissections (4 points) (=19 points) |
| **Group 4 (high, score ≥20 points)** | • An abdominal artery rupture (10 points) and an intrathoracic artery rupture (10 points) (=20 points)<br>• A carotid-cavernous fistula (10 points), 5 symptomatic dissections (10 points), and 3 aneurysms (3 points) (=23 points)<br>• An aortic rupture (10 points), 2 lower or upper limb arterial ruptures (10 points), and 3 symptomatic dissections (6 points) (=26 points)<br>• Two abdominal artery ruptures (20 points), a lower or upper limb arterial rupture (5 points), and 2 symptomatic dissections (4 points) (=29 points) |

Online Table 3: Characteristics of those who died before being seen by the service

| Sex | Diagnosed vEDS | vEDS variant | Inherited? | Age deceased | Cause of death |
|---|---|---|---|---|---|
| Male | PM COL3A1 FDR | Group_III | Yes | 44 | aortic dissection |
| Male | PM COL3A1 OC | Group_I | unknown | 64 | aortic dissection |
| Male | COL3A1 result PM | Group_I | unknown | 15 | ruptured vena cava |
| Female | Clinical diagnosis PM, COL3A1 FDR | Group_I | No | 30 | dissected iliac artery in childbirth |
| Male | COL3A1 result PM | Group_I | Yes | 19 | seizures secondary to frontal lobe infarction |
| Female | COL3A1 result PM | Group_I | Yes | 35 | aortic dissection |
| Female | COL3A1 result PM | Group_I | unknown | 33 | aortic dissection 7 days post c-section |
| Male | PM COL3A1 OC | Group_I | Yes | 34 | ruptured renal artery |
| Male | COL3A1 result PM | Group_III | Yes | 55 | dissecting aortic aneurysm |
| Male | Clinical diagnosis COL3A1 OC | Group_I | Yes | 44 | dissecting thoracic aneurysm |
| Male | COL3A1 result PM | Group_I | Yes | 24 | coronary artery rupture |
| Female | COL3A1 result PM | Group_I | unknown | 40 | died during heart valve surgery |
| Male | PM COL3A1 OC | Group_I | unknown | 34 | right common iliac arterial dissection |
| Male | COL3A1 result PM | Group_I | Yes | 18 | Dissection of whole length of aorta |
| Male | PM COL3A1 OC | Group_II | Yes | 37 | ruptured right renal artery aneurysm |
| Female | Clinical diagnosis COL3A1 in FDR | Group_II | unknown | 30 | abdominal hemorrhage |
| Male | PM COL3A1 OC | Group_I | unknown | 57 | dissecting thoracic aortic aneurysm |
| Male | COL3A1 test, result reported PM | Group_I | unknown | 31 | bleed into abdomen |
| Male | PM COL3A1 OC | Group_I | Yes | 43 | aortic dissection |

PM=post mortem diagnosis, OC=obligate carrier given results in the family, FDR=result in first degree relative

Online Table 4: showing *COL3A1* inheritance for probands and relatives

| inherited_dum | Index case or relative: Relative | Index | Total |
|---|---|---|---|
| unknown | 8<br>14.29 | 18<br>25.71 | 26<br>20.63 |
| No | 0<br>0.00 | 14<br>20.00 | 14<br>11.11 |
| Yes | 48<br>85.71 | 38<br>54.29 | 86<br>68.25 |
| Total | 56<br>100.00 | 70<br>100.00 | 126<br>100.00 |

Online Table 5: transitions in treatment from baseline to end of study

| Key |
|---|
| *frequency*<br>*row percentage* |

| basetreat | endtreat: ARB | BB | BB&ARB | celi | celi&ARB | no treatm | Total |
|---|---|---|---|---|---|---|---|
| ARB | 0<br>0.00 | 0<br>0.00 | 2<br>100.00 | 0<br>0.00 | 0<br>0.00 | 0<br>0.00 | 2<br>100.00 |
| BB | 0<br>0.00 | 0<br>0.00 | 6<br>100.00 | 0<br>0.00 | 0<br>0.00 | 0<br>0.00 | 6<br>100.00 |
| BB&ARB | 0<br>0.00 | 0<br>0.00 | 6<br>100.00 | 0<br>0.00 | 0<br>0.00 | 0<br>0.00 | 6<br>100.00 |
| celi | 1<br>7.14 | 0<br>0.00 | 3<br>21.43 | 2<br>14.29 | 8<br>57.14 | 0<br>0.00 | 14<br>100.00 |
| no treatm | 17<br>17.35 | 9<br>9.18 | 56<br>57.14 | 0<br>0.00 | 1<br>1.02 | 15<br>15.31 | 98<br>100.00 |
| Total | 18<br>14.29 | 9<br>7.14 | 73<br>57.94 | 2<br>1.59 | 9<br>7.14 | 15<br>11.90 | 126<br>100.00 |

Online Table 6: Evolution of the arterial score

| RECODE of fscore (fscore) | FSprogendFU very low | low | medium | high | Total |
|---|---|---|---|---|---|
| very low | 78<br>88.64 | 3<br>3.41 | 7<br>7.95 | 0<br>0.00 | 88<br>100.00 |
| low | 0<br>0.00 | 23<br>88.46 | 3<br>11.54 | 0<br>0.00 | 26<br>100.00 |
| medium | 0<br>0.00 | 0<br>0.00 | 9<br>81.82 | 2<br>18.18 | 11<br>100.00 |
| high | 0<br>0.00 | 0<br>0.00 | 0<br>0.00 | 1<br>100.00 | 1<br>100.00 |
| Total | 78<br>61.90 | 26<br>20.63 | 19<br>15.08 | 3<br>2.38 | 126<br>100.00 |

Online Table 7: Evolution of the arterial score by treatment at end of follow up

Treatment: ARB

Key: *frequency* / *row percentage*

| RECODE of fscore (fscore) | FSprogendFU very low | low | medium | Total |
|---|---|---|---|---|
| very low | 14<br>93.33 | 0<br>0.00 | 1<br>6.67 | 15<br>100.00 |
| low | 0<br>0.00 | 3<br>100.00 | 0<br>0.00 | 3<br>100.00 |
| Total | 14<br>77.78 | 3<br>16.67 | 1<br>5.56 | 18<br>100.00 |

Treatment: BB

Key: *frequency* / *row percentage*

| RECODE of fscore (fscore) | FSprogendFU very low | low | Total |
|---|---|---|---|
| very low | 7<br>87.50 | 1<br>12.50 | 8<br>100.00 |
| low | 0<br>0.00 | 1<br>100.00 | 1<br>100.00 |
| Total | 7<br>77.78 | 2<br>22.22 | 9<br>100.00 |

Treatment: BB&ARB

Key: *frequency* / *row percentage*

| RECODE of fscore (fscore) | FSprogendFU very low | low | medium | high | Total |
|---|---|---|---|---|---|
| very low | 43<br>91.49 | 2<br>4.26 | 2<br>4.26 | 0<br>0.00 | 47<br>100.00 |
| low | 0<br>0.00 | 15<br>93.75 | 1<br>6.25 | 0<br>0.00 | 16<br>100.00 |
| medium | 0<br>0.00 | 0<br>0.00 | 8<br>88.89 | 1<br>11.11 | 9<br>100.00 |
| high | 0<br>0.00 | 0<br>0.00 | 0<br>0.00 | 1<br>100.00 | 1<br>100.00 |
| Total | 43<br>58.90 | 17<br>23.29 | 11<br>15.07 | 2<br>2.74 | 73<br>100.00 |

Treatment: celi

Key: *frequency* / *row percentage*

| RECODE of fscore (fscore) | FSprogendFU very low | medium | Total |
|---|---|---|---|
| very low | 1<br>50.00 | 1<br>50.00 | 2<br>100.00 |
| Total | 1<br>50.00 | 1<br>50.00 | 2<br>100.00 |

Treatment: celi&ARB

Key: *frequency* / *row percentage*

| RECODE of fscore (fscore) | FSprogendFU very low | low | medium | Total |
|---|---|---|---|---|
| very low | 4<br>100.00 | 0<br>0.00 | 0<br>0.00 | 4<br>100.00 |
| low | 0<br>0.00 | 3<br>75.00 | 1<br>25.00 | 4<br>100.00 |
| medium | 0<br>0.00 | 0<br>0.00 | 1<br>100.00 | 1<br>100.00 |
| Total | 4<br>44.44 | 3<br>33.33 | 2<br>22.22 | 9<br>100.00 |

Treatment: no treatm

Key: *frequency* / *row percentage*

| RECODE of fscore (fscore) | FSprogendFU very low | low | medium | high | Total |
|---|---|---|---|---|---|
| very low | 9<br>75.00 | 0<br>0.00 | 3<br>25.00 | 0<br>0.00 | 12<br>100.00 |
| low | 0<br>0.00 | 1<br>50.00 | 1<br>50.00 | 0<br>0.00 | 2<br>100.00 |
| medium | 0<br>0.00 | 0<br>0.00 | 0<br>0.00 | 1<br>100.00 | 1<br>100.00 |
| Total | 9<br>60.00 | 1<br>6.67 | 4<br>26.67 | 1<br>6.67 | 15<br>100.00 |

Online Table 8: Characteristics of patients who died compared to surviving patients.

| Characteristics | Alive | Deceased* | p-value |
|---|---|---|---|
| n | 116 | 8 | |
| Sex | | | 0.71 |
| Female | 71(61.21) | 4(50) | |
| Male | 45(38.79) | 4(50) | |
| Age at start of FU | 33(20,45) | 24(16,26.75) | 0.07 |
| Age at end of FU | 37(25,50) | 26(19.25,30.5) | 0.05 |
| Status | | | 0.46 |
| Relative | 52(44.83) | 2(25) | |
| Proband | 64(55.17) | 6(75) | |
| Type of variant | | | 0.85 |
| Group I | 83(71.55) | 7(87.5) | |
| Group II | 20(17.24) | 1(12.5) | |
| Group III | 13(11.21) | 0(0) | |

Note: Values are n(%) or median (interquartile range). Fisher exact test for qualitative variables and Kruskal-Wallis test for quantitative variables.
*Excludes two patients who died of vEDS unrelated causes

Online Table 9: Distribution of vascular events in vEDS patients in full cohort.

| Type and location of vascular events | Number of vascular events | Number of patients |
|---|---|---|
| **All vascular events** | 165 | 72 (40%) |
| | 9 | 1 |
| | 7 | 2 |
| | 6 | 1 |
| | 5 | 4 |
| | 4 | 5 |
| | 3 | 9 |
| | 2 | 14 |
| | 1 | 36 |
| **Dissections** | 75 (45.45%) | 49 (27.22%)<br>(age range 13 to 65 years) |
| aortic | 12 | |
| arm | 2 | |
| carotid | 13 | |
| coeliac | 3 | |
| coronary | 7 | |
| femoral | 3 | |
| gastrointestinal | 2 | |
| hepatic | 1 | |
| iliac | 18 | |
| leg | 1 | |
| renal | 7 | |
| splenic | 1 | |
| thoracic aorta | 4 | |
| vertebral | 1 | |
| **Ruptures** | 22 (13.33%) | 17 (9.44%)<br>(age range 12 to 51 years) |
| arm | 3 | |
| cerebral | 1 | |
| coronary | 2 | |
| gastrointestinal | 5 | |
| iliac | 1 | |
| leg | 1 | |
| rectus sheath | 2 | |
| renal | 3 | |
| splenic | 2 | |
| superior vena cava | 1 | |
| thoracic aorta | 1 | |
| **Aneurysms** | 61 (36.96%) | 28 (15.55%)<br>(age range 17 to 63 years) |
| aortic | 2 | |
| arm | 3 | |
| carotid | 6 | |
| cavernous carotid | 1 | |
| cerebral | 1 | |
| coeliac | 8 | |
| coronary | 2 | |
| femoral | 3 | |
| gastrointestinal | 3 | |
| hepatic | 3 | |
| iliac | 9 | |
| leg | 2 | |
| renal | 9 | |
| splenic | 6 | |
| thoracic aorta | 1 | |
| vertebral | 2 | |
| **Carotid cavernous fistula** | 6 (3.63%) | 6 (3.33%)<br>(age range 29 to 55 years) |
| **Arteriovenous fistula** | 1 (0.60%) | 1 (0.55%)<br>(age 43 years) |

Online Figure 1: image of UK NHS vEDS emergency information card for medical professionals.

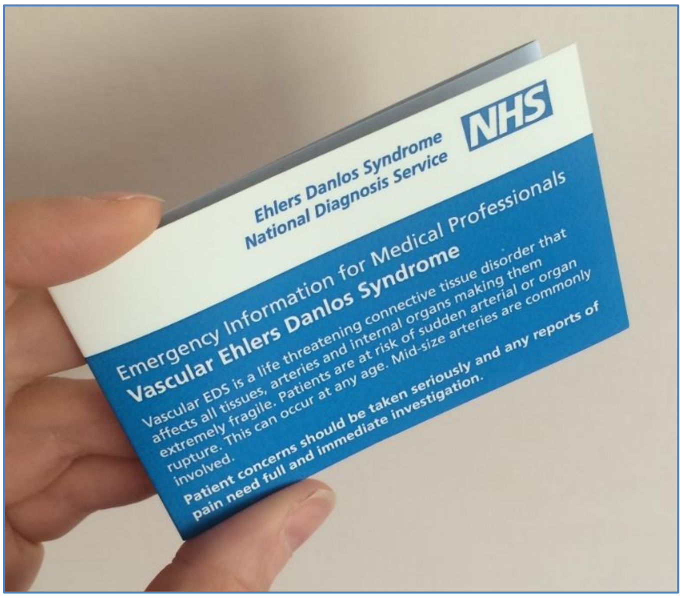


Online Figure 2. Showing range and distribution of BB and ARB used by cohort.

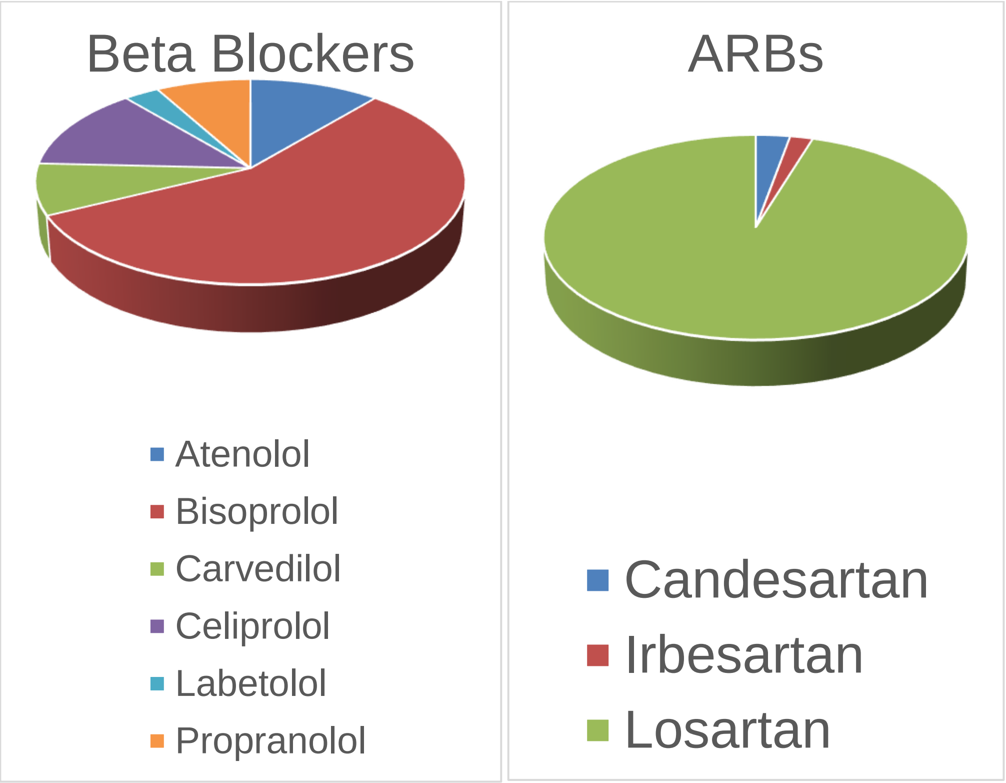

Online Figure 3: Showing peak ages for bowel perforation

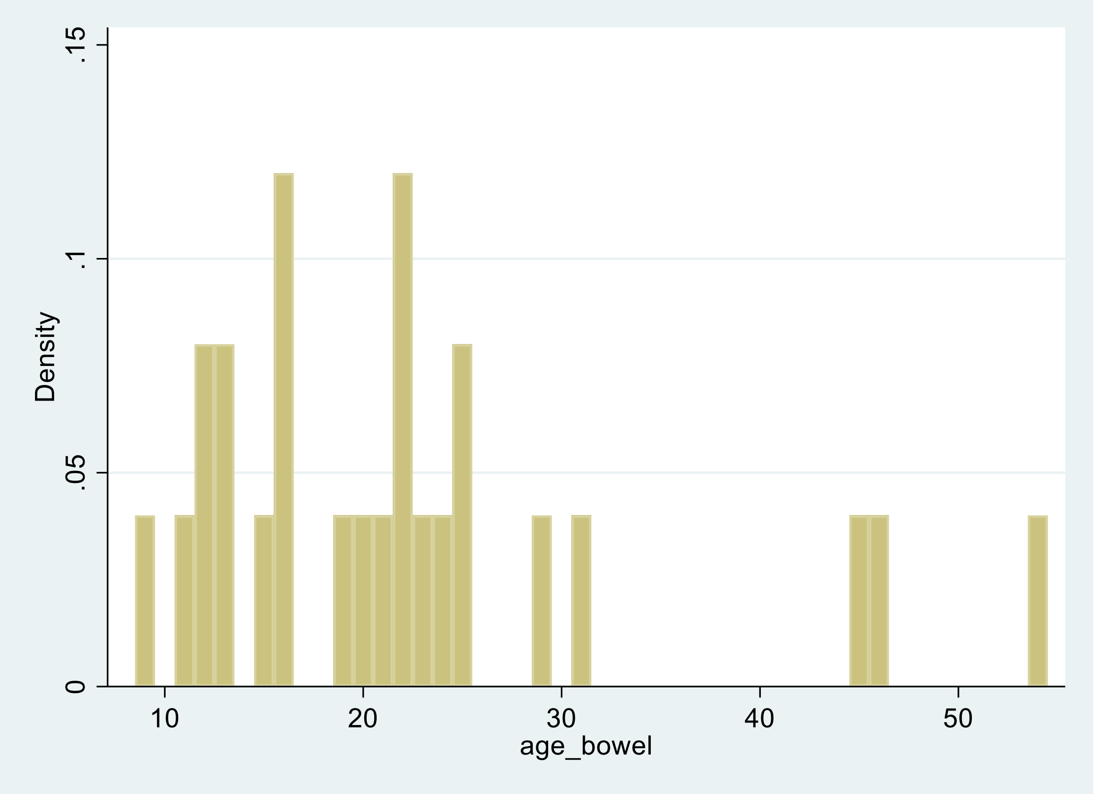

Online Figure 4. UK National Diagnostic Service Vascular EDS Emergency Information for Medical Professionals.

Ehlers Danlos Syndrome
National Diagnosis Service
NHS

# Emergency Information for Medical Professionals
## Vascular Ehlers Danlos Syndrome

Vascular EDS is a life threatening connective tissue disorder that affects all tissues, arteries and internal organs making them extremely fragile. Patients are at risk of sudden arterial or organ rupture. This can occur at any age. Mid-size arteries are commonly involved.
**Patient concerns should be taken seriously and any reports of pain need full and immediate investigation.**

**Presenting symptoms**
- Arterial or intestinal rupture commonly presents as sudden acute abdominal, chest or pelvic pain that can be diffuse or localised.
- Cerebral arterial rupture may present with altered mental status and be mistaken for drug or alcohol use.
- Redness, pain and prominence of one or both eyes and the sound of pulsations in the head can be manifestations of a carotid-cavernous fistula.
- Coronary dissection may present with acute myocardial infarction. Carotid dissection may present with stroke. Limb arterial dissection can present with acute limb ischaemia or claudication.
- Colonic perforation can present with acute abdominal pain and/or signs of infection.
- Pneumothorax can present with pain and shortness of breath.

**Management guidance**
The fragility of all tissues means that invasive procedures should be avoided where possible. All members of the medical team should be aware of the potential risk for greater than usual harm.
- Vascular dissection and rupture or bleeding can be subtle in presentation, therefore a lower threshold for investigations and imaging is indicated.
- Immediate investigation by MRI or CT scan should be performed.
- Use non-invasive techniques only, avoiding stress and tension on skin, organs or vessels during physical examination.
- Avoid angiography, enemas and endoscopies.
- Avoid intramuscular or subcutaneous injections of heparin or heparin substitutes, as these can cause massive subcutaneous haematoma and bruising.
- Central lines should be placed only with ultra sound guidance to avoid inadvertent arterial injuries.
- Fluoroquinolones/quinolones are a group of antibiotics that should be avoided if possible.

**Emergency surgery**
Surgical risks are higher for Vascular EDS patients. The threshold for intervention should be higher. All conservative management options should be carefully considered before surgery.
- The primary indication for surgical intervention is life threatening complications of arterial or organ rupture.
- A vascular surgeon should be present during surgery.
- The anaesthetist should be aware of fragile mucus membranes when intubating.
- Self retaining retractors should be used carefully, excessive retraction leads to multiple tissue tears and haematomas.
- Tissues are fragile and do not hold sutures well.

**Signs and symptoms that require medical attention**
- Severe pain in the head, neck or abdomen which may be diffuse or localised.
- Sudden onset of bleeding.
- Unexplained swelling of a limb.
- Symptoms of a stroke (drooping of the face, arm weakness, slurred or unclear speech).
- Weakness of the limbs.
- Dizziness or loss of consciousness.
- Shortness of breath and/or difficulty breathing or speaking.
- Visual disturbance.
- Redness, pain and prominence of one or both eyes.
- The sound of pulsations in the head.
- A large bruise increasing in size.

**Vascular EDS is a serious condition that is associated with a risk of sudden arterial or organ rupture at any age.**

Version 3. Review date: 01/07/2023

Online Figure 5. Poster Abstract 38, International Scientific Symposium on EDS & HSD, 2022

NHS
Ehlers Danlos Syndrome
National Diagnostic Service

38

# Vascular EDS and Pregnancy

**Jessica M Bowen**[1], Diana S Johnson[1], Tessa Bonnett[2], Laura Hammon[2], Ruth Newton[2], Claire Green[1], Tammy Kammin[1], Naomi Taylor[3], Oliver Watson[3], Nigel M Wheeldon[3], Glenda J Sobey[1].
[1]Ehlers-Danlos Syndrome National Diagnostic Service, Sheffield Children's Hospital NHS Foundation Trust, OPD2 Department, Northern General Hospital, Sheffield, S5 7AU. [2]Jessop Wing, Sheffield Teaching Hospitals NHS Foundation Trust, Royal Hallamshire Hospital, Sheffield, S10 2JF. [3] South Yorkshire Regional Inherited Cardiac Conditions Service, Sheffield Teaching Hospitals NHS Foundation Trust, Northern General Hospital, Sheffield, S5 7AU.

## Background

The UK EDS Diagnostic service was established in 2009 to improve diagnosis for people with the rare types of EDS. The service has two clinic locations; Sheffield and London. Over 200 patients diagnosed with vascular EDS (vEDS) have now been seen by the team in Sheffield. The service has been involved in the care of women whose diagnosis of vEDS has been made during pregnancy, before pregnancy and women who had pregnancies before knowing their diagnosis.

## Discussion with patient

We talk to our patients about the risks associated with pregnancy for women with vEDS as well as the 50% chance of a child inheriting the condition if either parent has the diagnosis. We discuss reproductive options, including prenatal diagnoses, preimplantation genetic testing (PGT), surrogacy and adoption with both male and female patients who may be considering a family. We give specific advice on the associated risk of each option for women with vascular EDS. Pregnancy is known to carry significant risks for women with vEDS (1). All women with vEDS are classified as high-risk in terms of pregnancy management (2) and need carefully coordinated care under specialist obstetric services in tertiary care. Prior history of vascular events of hollow organ rupture would further increase these risks.

## Reproductive options

- Antenatal diagnosis by chorionic villus sampling (CVS) between 11-14 weeks of pregnancy or amniocentesis between 15-20 weeks of pregnancy may be considered by women with vEDS. These procedures carry a risk of miscarriage for all women (up to 1%). Additional risks for women with vEDS are unknown but expected given the increased tissue fragility. The timing of the procedure should be considered, with the option of admitting the patient, rather than managing as an outpatient.
- Termination of pregnancy may be considered by women with vEDS. The additional risks of termination of pregnancy for women with vEDS are unknown. Medical termination would be advised rather than surgical termination due to blood vessel and tissue fragility and bleeding risks.
- Egg donation with surrogacy may be considered by women with vEDS not wanting the risks associated with carrying a pregnancy. For women choosing to use their own egg the additional risks for women with vEDS related to ovarian hyperstimulation and egg retrieval are unknown.
- Preimplantation genetic testing (PGT) may be considered by women with vEDS and may be offered depending on medical history. Additional risks for women with vEDS are unknown but include potential risks from ovarian hyperstimulation and egg retrieval, as well as risks associated with pregnancy.
- Adoption/Fostering may be considered by women with vEDS to avoid the risks associated with pregnancy. A welfare of the child assessment will include consideration of the woman's vEDS diagnosis and statements from specialists involved are likely to be requested.

## Prior to pregnancy

Beta-blockers and Losartan are often used in the management of vEDS. Losartan is contraindicated in pregnancy; this should be stopped, ideally before pregnancy. Beta-blockers may need to be changed (to alternative beta blocker labetalol) during pregnancy. Beta-blockers carry a small risk of fetal growth restriction so serial fetal growth scans should be arranged.

Where possible, MRA scans should be performed prior to pregnancy and should be reviewed with plans for further imaging based on these results. Scans in pregnancy may be needed if no previous scans are available. Postpartum vascular imaging is recommended at 3 months.

## Pregnancy timeline

Discussion before pregnancy
- Prior risks
- Reproductive options
- Contraception
- Antenatal options
- Vascular imaging
- Medication

Discussion in 1st Trimester
- Medical team
- Antenatal options
- Medication

Discussion in 2nd Trimester
- Plans for delivery
- Obstetric anaesthetic assessment
- Plans following delivery
- Consider cord blood storage
- Fetal growth scans

3rd Trimester
- Antenatal steroid injection before delivery
- Ensure cross matched blood available

Delivery
- Elective caesarean section
- Vascular surgeon available
- 34/35 weeks
- MOH protocol
- Cord blood storage if requested
- Consider contraceptive options

Postpartum
- Observe mother in obstetric HDU or critical care unit
- Neonatal team to be aware of potential vEDS diagnosis in baby
- Consider medication and vascular imaging for mother

## Pregnancy management for women with vEDS

The Sheffield EDS Diagnostic Service have now been involved with the successfully delivery of 12 babies to women with known diagnosis of vEDS. These pregnancies were closely managed by a specialist obstetric team in a tertiary unit with MDT involvement including; obstetrics, anaesthetists, vascular surgeons, clinical genetics, cardiology (including cardiologist with special knowledge of vEDS) and input from the EDS Diagnostic Service.

Early elective caesarean section with delivery suggested at 34/35 weeks to balance the risks to the baby of early delivery with the risks of spontaneous labour for the mother. There is an increased risk of premature birth for babies with vEDS and an increased risk of PROM for mothers with vEDS. Antenatal steroid administration for fetal lung maturation is recommended between 24 hours and 7 days prior to delivery.

An obstetric anaesthetic assessment should take place in the antenatal period. A specialist obstetric anaesthetist is required for delivery and the method of anaesthesia needs to be carefully considered. A vascular surgeon (and appropriate surgical equipment) should be available in theatre for immediate expert repair of vessels if needed. Protocolised management of Massive Obstetric Haemorrhage (MOH) should be readily accessible for those patients that bleed; ideally, as well as cross-matched blood, use of cell salvage, bedside monitoring of clotting function (to guide blood product administration), and fibrinogen concentrate.

Parents may wish to have cord blood stored at birth to allow genetic testing at a later date. It is helpful to have a request form completed for the parents to hand over before delivery.

Some women with vEDS may wish to consider the option of having a tubal ligation at the time of delivery. Other contraceptive options can be discussed. Although it is possible to fit a coil under direct vision at c-section, replacement carries risk as it is not performed under direct vision and there is an increased risk of uterine perforation or coil migration

Mother – to be observed in obstetric HDU or critical care unit overnight with critical care team aware. Consider extending the postnatal stay in hospital as vessel dissection and rupture in the mother are reported also in the postnatal period.

## Pregnancy management for partners of men with vEDS

Men with vEDS have the same reproductive options as women. There is a 50% chance of a baby inheriting vEDS and in the absence of genetic testing the potential for preterm delivery should be recognised. Parents may wish to have cord blood stored at birth to allow genetic testing at a later date. It is helpful to have a request form completed for the parents to hand over before delivery

## Neonate at 50% risk of having vEDS

The neonatal team needs to be aware of the risk of vEDS in the baby and potential complications in an affected neonate. It is not possible to make a confident clinical diagnosis of vEDS in a neonate. Talipes is seen more commonly in babies with vEDS. There are cases of pneumothorax reported in the early neonatal period if the baby is affected with vEDS.

There are currently no international guidelines for the management of pregnancy for women with vEDS. By sharing our experience of successfully managed pregnancies and deliveries we hope to be able to improve the outcomes for women with vEDS in pregnancy.

References:
1) MURRAY, M.L., PEPIN, M., PETERSON, S. and BYERS, P.H., 2014. Pregnancy-related deaths and complications in women with vascular Ehlers–Danlos syndrome. *Genetics in medicine*, **16**(12), pp. 874-880.
2) 2018 ESC Guidelines for the management of cardiovascular diseases during pregnancy. European Heart Journal (2018) 39, 3165–3241

Online Figure 6: Additional survival curves

a) All treatment groups: log=rank test for equality of survivor functions. p-value =0.00

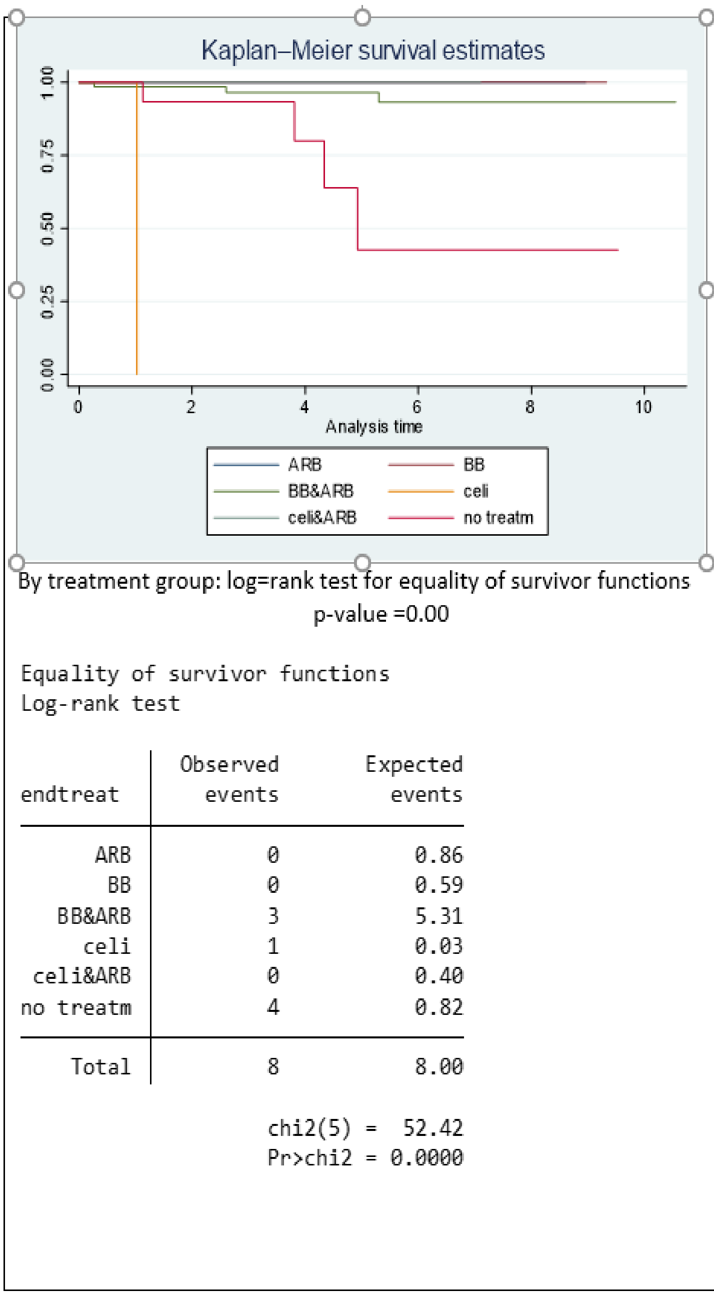


By treatment group: log=rank test for equality of survivor functions p-value =0.00

Equality of survivor functions
Log-rank test

| endtreat | Observed events | Expected events |
|---|---|---|
| ARB | 0 | 0.86 |
| BB | 0 | 0.59 |
| BB&ARB | 3 | 5.31 |
| celi | 1 | 0.03 |
| celi&ARB | 0 | 0.40 |
| no treatm | 4 | 0.82 |
| Total | 8 | 8.00 |

chi2(5) = 52.42
Pr>chi2 = 0.0000

aplan-Meier survivor function
y variable: endtreat

| Time | Beg. total | Fail | Survivor function | Std. error | [95% conf. | int.] |
|---|---|---|---|---|---|---|
| ARB | | | | | | |
| 1 | 17 | 0 | 1.0000 | . | . | . |
| 2 | 12 | 0 | 1.0000 | . | . | . |
| 3 | 8 | 0 | 1.0000 | . | . | . |
| 4 | 6 | 0 | 1.0000 | . | . | . |
| 5 | 5 | 0 | 1.0000 | . | . | . |
| 6 | 3 | 0 | 1.0000 | . | . | . |
| 7 | 2 | 0 | 1.0000 | . | . | . |
| 8 | 2 | 0 | 1.0000 | . | . | . |
| 9 | 1 | 0 | . | . | . | . |
| 10 | 1 | 0 | . | . | . | . |
| 11 | 1 | 0 | . | . | . | . |
| BB | | | | | | |
| 1 | 0 | 0 | 1.0000 | . | . | . |
| 2 | 6 | 0 | 1.0000 | . | . | . |
| 3 | 5 | 0 | 1.0000 | . | . | . |
| 4 | 5 | 0 | 1.0000 | . | . | . |
| 5 | 5 | 0 | 1.0000 | . | . | . |
| 6 | 3 | 0 | 1.0000 | . | . | . |
| 7 | 3 | 0 | 1.0000 | . | . | . |
| 8 | 2 | 0 | 1.0000 | . | . | . |
| 9 | 2 | 0 | 1.0000 | . | . | . |
| 10 | 1 | 0 | . | . | . | . |
| 11 | 1 | 0 | . | . | . | . |
| BB&ARB | | | | | | |
| 1 | 67 | 1 | 0.9853 | 0.0146 | 0.9002 | 0.9979 |
| 2 | 57 | 0 | 0.9853 | 0.0146 | 0.9002 | 0.9979 |
| 3 | 48 | 1 | 0.9652 | 0.0245 | 0.8656 | 0.9913 |
| 4 | 39 | 0 | 0.9652 | 0.0245 | 0.8656 | 0.9913 |
| 5 | 34 | 0 | 0.9652 | 0.0245 | 0.8656 | 0.9913 |
| 6 | 27 | 1 | 0.9330 | 0.0395 | 0.7949 | 0.9793 |
| 7 | 18 | 0 | 0.9330 | 0.0395 | 0.7949 | 0.9793 |
| 8 | 6 | 0 | 0.9330 | 0.0395 | 0.7949 | 0.9793 |
| 9 | 4 | 0 | 0.9330 | 0.0395 | 0.7949 | 0.9793 |
| 10 | 2 | 0 | 0.9330 | 0.0395 | 0.7949 | 0.9793 |
| 11 | 1 | 0 | . | . | . | . |
| celi | | | | | | |
| 1 | 0 | 0 | 1.0000 | . | . | . |
| 2 | 1 | 1 | . | . | . | . |
| 3 | 1 | 0 | . | . | . | . |
| 4 | 1 | 0 | . | . | . | . |
| 5 | 1 | 0 | . | . | . | . |
| 6 | 1 | 0 | . | . | . | . |
| 7 | 1 | 0 | . | . | . | . |
| 8 | 1 | 0 | . | . | . | . |
| 9 | 1 | 0 | . | . | . | . |
| 10 | 1 | 0 | . | . | . | . |
| 11 | 1 | 0 | . | . | . | . |
| celi&ARB | | | | | | |
| 1 | 0 | 0 | 1.0000 | . | . | . |
| 2 | 8 | 0 | 1.0000 | . | . | . |
| 3 | 4 | 0 | 1.0000 | . | . | . |
| 4 | 3 | 0 | 1.0000 | . | . | . |
| 5 | 2 | 0 | 1.0000 | . | . | . |
| 6 | 2 | 0 | 1.0000 | . | . | . |
| 7 | 2 | 0 | 1.0000 | . | . | . |
| 8 | 1 | 0 | . | . | . | . |
| 9 | 1 | 0 | . | . | . | . |
| 10 | 1 | 0 | . | . | . | . |
| 11 | 1 | 0 | . | . | . | . |
| no treatm | | | | | | |
| 1 | 0 | 0 | 1.0000 | . | . | . |
| 2 | 14 | 1 | 0.9333 | 0.0644 | 0.6126 | 0.9903 |
| 3 | 10 | 0 | 0.9333 | 0.0644 | 0.6126 | 0.9903 |
| 4 | 7 | 1 | 0.8000 | 0.1352 | 0.3735 | 0.9507 |
| 5 | 3 | 2 | 0.4267 | 0.2113 | 0.0698 | 0.7614 |
| 6 | 2 | 0 | 0.4267 | 0.2113 | 0.0698 | 0.7614 |
| 7 | 2 | 0 | 0.4267 | 0.2113 | 0.0698 | 0.7614 |
| 8 | 2 | 0 | 0.4267 | 0.2113 | 0.0698 | 0.7614 |
| 9 | 2 | 0 | 0.4267 | 0.2113 | 0.0698 | 0.7614 |
| 10 | 1 | 0 | . | . | . | . |
| 11 | 1 | 0 | . | . | . | . |

Note: Survivor function is calculated over full data and evaluated at indicated times; it is not calculated from aggregates shown at left.

b) By treatment group with celiprolol and celiprolol&ARB groups merged

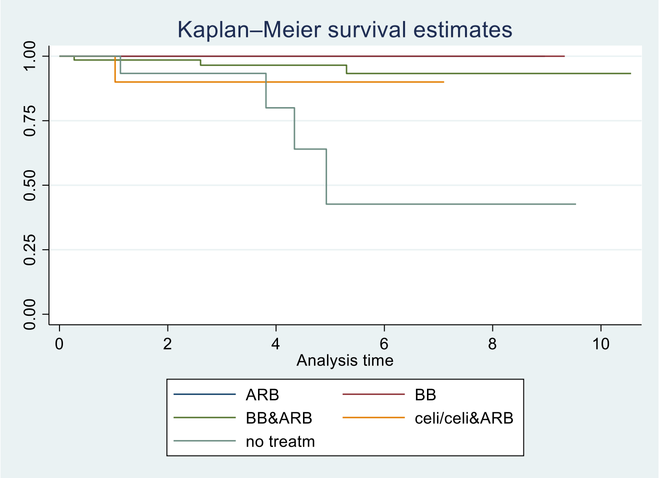


Equality of survivor functions
Log-rank test

| fiveendtreat | Observed events | Expected events |
|---|---|---|
| ARB | 0 | 0.86 |
| BB | 0 | 0.59 |
| BB&ARB | 3 | 5.31 |
| celi/celi&ARB | 1 | 0.42 |
| no treatm | 4 | 0.82 |
| Total | 8 | 8.00 |

chi2(4) = 15.79
Pr>chi2 = 0.0033

Kaplan-Meier survivor function
By variable: fiveendtreat

| Time | Beg. total | Fail | Survivor function | Std. error | [95% conf. int.] | |
|---|---|---|---|---|---|---|
| ARB | | | | | | |
| 1 | 17 | 0 | 1.0000 | . | . | . |
| 2 | 12 | 0 | 1.0000 | . | . | . |
| 3 | 8 | 0 | 1.0000 | . | . | . |
| 4 | 6 | 0 | 1.0000 | . | . | . |
| 5 | 5 | 0 | 1.0000 | . | . | . |
| 6 | 3 | 0 | 1.0000 | . | . | . |
| 7 | 2 | 0 | 1.0000 | . | . | . |
| 8 | 2 | 0 | 1.0000 | . | . | . |
| 9 | 1 | 0 | . | . | . | . |
| 10 | 1 | 0 | . | . | . | . |
| 11 | 1 | 0 | . | . | . | . |
| BB | | | | | | |
| 1 | 0 | 0 | 1.0000 | . | . | . |
| 2 | 6 | 0 | 1.0000 | . | . | . |
| 3 | 5 | 0 | 1.0000 | . | . | . |
| 4 | 5 | 0 | 1.0000 | . | . | . |
| 5 | 5 | 0 | 1.0000 | . | . | . |
| 6 | 3 | 0 | 1.0000 | . | . | . |
| 7 | 3 | 0 | 1.0000 | . | . | . |
| 8 | 2 | 0 | 1.0000 | . | . | . |
| 9 | 2 | 0 | 1.0000 | . | . | . |
| 10 | 1 | 0 | . | . | . | . |
| 11 | 1 | 0 | . | . | . | . |
| BB&ARB | | | | | | |
| 1 | 67 | 1 | 0.9853 | 0.0146 | 0.9002 | 0.9979 |
| 2 | 57 | 0 | 0.9853 | 0.0146 | 0.9002 | 0.9979 |
| 3 | 48 | 1 | 0.9652 | 0.0245 | 0.8656 | 0.9913 |
| 4 | 39 | 0 | 0.9652 | 0.0245 | 0.8656 | 0.9913 |
| 5 | 34 | 0 | 0.9652 | 0.0245 | 0.8656 | 0.9913 |
| 6 | 27 | 1 | 0.9330 | 0.0395 | 0.7949 | 0.9793 |
| 7 | 18 | 0 | 0.9330 | 0.0395 | 0.7949 | 0.9793 |
| 8 | 6 | 0 | 0.9330 | 0.0395 | 0.7949 | 0.9793 |
| 9 | 4 | 0 | 0.9330 | 0.0395 | 0.7949 | 0.9793 |
| 10 | 2 | 0 | 0.9330 | 0.0395 | 0.7949 | 0.9793 |
| 11 | 1 | 0 | . | . | . | . |
| celi/celi&ARB | | | | | | |
| 1 | 0 | 0 | 1.0000 | . | . | . |
| 2 | 8 | 1 | 0.9000 | 0.0949 | 0.4730 | 0.9853 |
| 3 | 4 | 0 | 0.9000 | 0.0949 | 0.4730 | 0.9853 |
| 4 | 3 | 0 | 0.9000 | 0.0949 | 0.4730 | 0.9853 |
| 5 | 2 | 0 | 0.9000 | 0.0949 | 0.4730 | 0.9853 |
| 6 | 2 | 0 | 0.9000 | 0.0949 | 0.4730 | 0.9853 |
| 7 | 2 | 0 | 0.9000 | 0.0949 | 0.4730 | 0.9853 |
| 8 | 1 | 0 | . | . | . | . |
| 9 | 1 | 0 | . | . | . | . |
| 10 | 1 | 0 | . | . | . | . |
| 11 | 1 | 0 | . | . | . | . |
| no treatm | | | | | | |
| 1 | 0 | 0 | 1.0000 | . | . | . |
| 2 | 14 | 1 | 0.9333 | 0.0644 | 0.6126 | 0.9903 |
| 3 | 10 | 0 | 0.9333 | 0.0644 | 0.6126 | 0.9903 |
| 4 | 7 | 1 | 0.8000 | 0.1352 | 0.3735 | 0.9507 |
| 5 | 3 | 2 | 0.4267 | 0.2113 | 0.0698 | 0.7614 |
| 6 | 2 | 0 | 0.4267 | 0.2113 | 0.0698 | 0.7614 |
| 7 | 2 | 0 | 0.4267 | 0.2113 | 0.0698 | 0.7614 |
| 8 | 2 | 0 | 0.4267 | 0.2113 | 0.0698 | 0.7614 |
| 9 | 2 | 0 | 0.4267 | 0.2113 | 0.0698 | 0.7614 |
| 10 | 1 | 0 | . | . | . | . |
| 11 | 1 | 0 | . | . | . | . |

Note: Survivor function is calculated over full data and evaluated at indicated times; it is not calculated from aggregates shown at left.

c) Treated vs untreated excluding those with group III variant

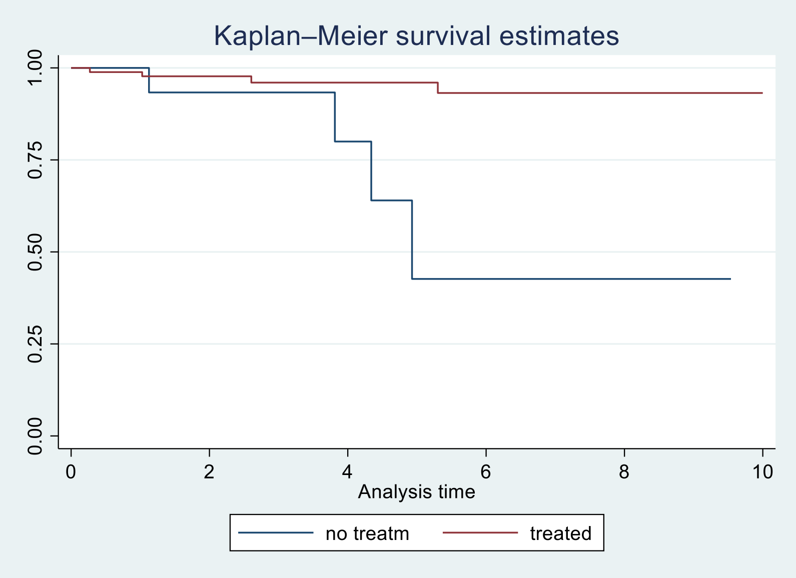


Equality of survivor functions
Log-rank test

| treatvsnot | Observed events | Expected events |
|---|---|---|
| no treatm | 4 | 0.92 |
| treated | 4 | 7.08 |
| Total | 8 | 8.00 |

chi2(1) = 11.87
Pr>chi2 = 0.0006

Kaplan-Meier survivor function
By variable: treatvsnot

| Time | Beg. total | Fail | Survivor function | Std. error | [95% conf. | int.] |
|---|---|---|---|---|---|---|
| no treatm | | | | | | |
| 1 | 0 | 0 | 1.0000 | . | . | . |
| 2 | 14 | 1 | 0.9333 | 0.0644 | 0.6126 | 0.9903 |
| 3 | 10 | 0 | 0.9333 | 0.0644 | 0.6126 | 0.9903 |
| 4 | 7 | 1 | 0.8000 | 0.1352 | 0.3735 | 0.9507 |
| 5 | 3 | 2 | 0.4267 | 0.2113 | 0.0698 | 0.7614 |
| 6 | 2 | 0 | 0.4267 | 0.2113 | 0.0698 | 0.7614 |
| 7 | 2 | 0 | 0.4267 | 0.2113 | 0.0698 | 0.7614 |
| 8 | 2 | 0 | 0.4267 | 0.2113 | 0.0698 | 0.7614 |
| 9 | 2 | 0 | 0.4267 | 0.2113 | 0.0698 | 0.7614 |
| 10 | 1 | 0 | . | . | . | . |
| 11 | 1 | 0 | . | . | . | . |
| treated | | | | | | |
| 1 | 89 | 1 | 0.9886 | 0.0113 | 0.9221 | 0.9984 |
| 2 | 72 | 1 | 0.9773 | 0.0159 | 0.9122 | 0.9943 |
| 3 | 54 | 1 | 0.9601 | 0.0231 | 0.8785 | 0.9873 |
| 4 | 45 | 0 | 0.9601 | 0.0231 | 0.8785 | 0.9873 |
| 5 | 40 | 0 | 0.9601 | 0.0231 | 0.8785 | 0.9873 |
| 6 | 29 | 1 | 0.9319 | 0.0357 | 0.8150 | 0.9760 |
| 7 | 19 | 0 | 0.9319 | 0.0357 | 0.8150 | 0.9760 |
| 8 | 6 | 0 | 0.9319 | 0.0357 | 0.8150 | 0.9760 |
| 9 | 4 | 0 | 0.9319 | 0.0357 | 0.8150 | 0.9760 |
| 10 | 1 | 0 | 0.9319 | 0.0357 | 0.8150 | 0.9760 |
| 11 | 1 | 0 | . | . | . | . |

Note: Survivor function is calculated over full data and evaluated at indicated times; it is not calculated from aggregates shown at left.
